# An Epidemic of Respiratory and Ocular Infections Caused by the Reemergence of a Recombinant Human Adenovirus, the Novel Type HAdV‐B114 (P7H3F3)

**DOI:** 10.1002/jmv.70464

**Published:** 2025-06-30

**Authors:** Tina Ganzenmueller, Magnus Wolf, Lasse Wolfram, Charikleia Gkioule, Lars Steinbrück, Albert Heim

**Affiliations:** ^1^ Institute for Medical Virology, University Hospital Tuebingen Tuebingen Germany; ^2^ German Center for Infection Research (DZIF), Site Tuebingen Tuebingen Germany; ^3^ Department for Ophthalmology University Hospital Tuebingen Tuebingen Germany; ^4^ Department for Ophthalmology, Institute for Ophthalmic Research Eberhard Karls University of Tuebingen Tuebingen Germany; ^5^ Institute of Virology, Hannover Medical School Hannover Germany; ^6^ German National Adenovirus Reference Laboratory Hannover Germany

**Keywords:** conjunctivitis, high‐throughput sequencing, molecular typing, novel adenovirus, pneumonia

## Abstract

Human adenoviruses of species B (HAdV‐B) can cause upper respiratory tract infections and conjunctivitis, but also severe lower respiratory tract infections (LRTI). Although HAdV‐associated LRTI are non‐notifiable in Germany, typing data of our Adenovirus Reference Laboratory indicated an HAdV‐B3 epidemic in 2023, with 67 samples initially typed as HAdV‐B3 compared to < 10/year in the previous years. Circulation of a novel, highly virulent HAdV‐B3 strain was suspected and complete viral genomic sequencing performed, revealing a recombinant phylogeny of the penton gene (P), which originated from HAdV‐B7, whereas hexon (H) and fiber (F) genes originated from HAdV‐B3. Therefore, this virus was acknowledged by the Adenovirus Working Group as the novel recombinant genotype 114 (P7H3F3). Interestingly, BLAST search of the HAdV‐B114 prototype sequence showed 99.91% identity to the old HAdV‐B genome type 3a. Additionally, multiple complete adenovirus genomic sequences labeled as HAdV‐B3 during the last two decades had > 99.8% identity, suggesting long‐term circulation of HAdV‐B114 although the recombinant phylogeny of its penton region had not been recognized. This detailed analysis of an HAdV epidemic associated with ocular and respiratory infections, including severe LRTI, led to the discovery of a novel genotype HAdV‐B114, which is rather a neglected, re‐emergent than an emerging virus.

**Abbreviations**
BAL: bronchioalveolar lavage
HAdV: human adenovirus
HSCT: hematopoietic stem cell transplantation
HTS: high‐throughput sequencing
LRTI: lower respiratory tract infection
PCF: pharyngoconjunctival fever
RFLP: restriction fragment length polymorphism

## 1 Background

Human adenoviruses (HAdV) are non‐enveloped DNA viruses and belong to the genus *Mastadenovirus* within the family Adenoviridae. HAdV types (up to type 51) were initially defined and typed by neutralization testing (“serotypes”). Sanger sequencing of the hexon gene (imputed serotyping by partial sequencing of the region encoding the neutralization epitope of the hexon capsid protein) is still an effective method in many diagnostic laboratories, but cannot correctly type novel, recombinant genotypes [1, 2, 3].

The multitude of HAdV types is classified into species HAdV A to G, initially based on hemagglutination properties and subsequently on molecular phylogeny. An important mechanism of adenovirus evolution is homologous recombination between types of the same HAdV species, which can lead to the emergence of novel HAdV types (“genotypes”) [4, 5]. With the advent of high‐throughput sequencing (HTS), adenovirus genotyping has been established based on three gene regions encoding the major viral capsid proteins: penton (P), hexon (H), and fiber (F) [2, 6]. This approach can be used for the discovery of new HAdV types (genotypes) that require confirmation by the Human Adenovirus Working Group, as well as for accurate diagnostic typing of all serotypes and genotypes.

HAdV species B includes (among others) HAdV‐3, ‐7, ‐14, ‐21, ‐55, and ‐66 sharing a tropism for the respiratory tract. These viruses cause infections of the upper respiratory tract infections (URTI), such as common cold and pharyngoconjunctival fever (PCF), but also more severe lower respiratory tract infections (LRTI), for example, pneumonia and acute respiratory distress syndrome (ARDS) even in immunocompetent patients [7, 8, 9]. HAdV‐B55 and HAdV‐B66 were associated with recent outbreaks of severe LRTIs but had been typed initially as HAdV‐B11 or HAdV‐B7, respectively, due to their recombinant phylogeny with neutralization epitope sequences of the latter viruses [1, 10, 11, 12, 13, 14]. Therefore, a recent epidemic of LRTIs and ocular infections in Germany caused by the rather endemic type HAdV‐B3, raised the suspicion of the emergence of a novel HAdV‐B genotype retaining a HAdV‐B3 neutralization epitope sequence in a recombinant genome which may result in enhanced virulence and transmissibility.

Here we describe clinical and phylogenetic features of this virus, which has been accepted by the Human Adenovirus Working Group (http://hadvwg.gmu.edu/) as the novel recombinant genotype HAdV‐B114.

## 2 Methods

### 2.1 Patients, Samples, and Study Design

In 2023, 79 HAdV‐B‐DNA‐positive specimens (including basic clinical information such as LRTI, PCF, or conjunctivitis) were provided by diagnostic laboratories from all over Germany for HAdV typing in our Adenovirus Reference Laboratory. In total, 85% (67/79) of these samples were typed by imputed serotyping as HAdV‐B3. These were investigated in more detail by complete genomic sequencing (or Sanger sequencing of fiber and penton genes if complete genomic sequencing failed). Specimen types included secretions/swabs from the upper or lower respiratory tract, bronchoalveolar lavages, eye swabs, blood, and stool. Medical data from a subset of LRTI and conjunctivitis patients infected with HAdV‐B114 (initially typed as HAdV‐B3) and treated at the University Hospital Tuebingen were collected to characterize the clinical features more detailed. In addition, archived DNA samples available from 31 of 48 HAdV‐B3 positive diagnostic specimens sampled between 2007 and 2021 were sequenced to determine whether HAdV‐B114 circulated before 2023.

This study was approved by the Ethics Committee of the University Hospital Tuebingen (No. 774/2023BO2).

### 2.2 Genotyping by Partial Sequencing of the Penton, Hexon, and Fiber Gene

Routine diagnostic molecular typing (imputed serotyping) was performed by partial hexon gene sequencing [15]. Briefly, HAdV‐DNA was amplified by conventional generic PCR targeting the sequence of the immunogenic loop 2 of the *ε* determinant of the hexon gene [16]. Subsequently, fiber and penton genes were sequenced as previously described [15, 17], if complete genomic sequencing was not feasible. Cycle sequencing (Applied Biosystems) was performed in both directions using the same primers as for PCR [17, 18].

### 2.3 Viral Genome Sequencing and De Novo Assembly

HTS of adenoviral genomes was performed as previously described [19]. Briefly, DNA was extracted from HAdV‐positive clinical specimens or cell culture isolates on A549 cells (Supporting Information S1: Table S1) on a QIAcube (Qiagen Blood Kit) and sequencing libraries were prepared using the NEBNext Ultra II FS DNA Library Prep Kit for sequencing on an Illumina MiSeq. After quality control of raw data, human reads were removed, the remaining reads were trimmed with fastp and de novo assembled with SPAdes, Minia3, or GATB tools [19]. After scaffolding of the genomes and several correction steps (Pilon and GATK), genome annotation was done using Geneious Prime. A more methodological description can be found in Supporting Information S1: Supplementary Data.

### 2.4 Phylogenetic Analysis

A multiple alignment of whole genome sequences was constructed by using fast Fourier transforms (MAFFT) (https://www.ebi.ac.uk/Tools/msa/mafft/) with default gap parameters. Pairwise alignment, comparisons, and visualization of genomes were performed on BioEdit version 7.2.0 (http://www.mbio.ncsu.edu/BioEdit/page2.html). Bootstrapped, maximum likelihood phylogenetic trees with 500 replicates were constructed using RAxML version 8.2.11 with the command line options: ‐m GTRGAMMA, ‐f a, ‐x 1, ‐N 500, ‐p 1. MEGA v12 software was used to visualize the trees. Similarity plots and recombination detection (bootscan approach) were performed using Simplot software (version 3.5.1) with a window size of 1000 bp and a step size of 200 bp. In addition to the novel HAdV‐B114 sequence, complete genomic nucleotide sequences representing all prototypes of the HAdV‐B species, genome type HAdV‐B3a and HAdV‐B7 vaccine strain were used in the analysis (GenBank accession numbers: OR853835, PQ189748, PQ189750, KF268123, JX423381, AY599834, JN860676, AY594255, MN936178, AY601636, JN860678, AY163756, KT970441, LC177352, ON393912, AY803294, FJ643676, AY601633, KF633445, AY737797, AY128640, KF268328, AY737798).

Virtual restriction fragment length polymorphism (RFLP) patterns for the complete genomic sequences of HAdV‐B114 and genome type 3A were generated using the online tool Restriction Analyzer (https://molbiotools.com/restrictionanalyzer.php).

## 3 Results

### 3.1 HAdV‐B3 Infections From 2012 to 2023 in Germany

**Figure 1:**
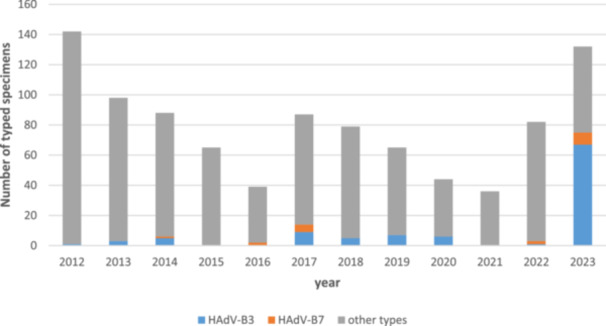
Overview of HAdV types detected in samples submitted to the German Adenovirus Reference Laboratory from 2012 to 2023. All typing results (based on partial HAdV hexon gene sequencing) have been summarized for each year. HAdV‐B3 and ‐B7 types are highlighted in blue and orange, respectively. All HAdV‐B3 typing results of 2023 have to be revised as HAdV‐B114 after penton base gene sequencing or complete genomic sequencing.

HAdV‐associated respiratory infections are a non‐notifiable disease in Germany but surveillance is voluntarily performed by diagnostic laboratories, which send samples of severe cases to the HAdV Reference Laboratory for HAdV typing by imputed serotyping. These results from 2012 to 2023 are provided in Figure 1. The observed “epidemic” of HAdV‐B3 (and to a far lesser extent of HAdV‐B7) in 2023 was associated with symptoms of severe LRTI, URTI, conjunctivitis and/or PCF as indicated on the typing requests. In addition, HAdV‐DNAemia and shedding in stool samples, suggesting disseminated infection, were observed in several patients (see also details of typical cases below). All specimens initially typed as HAdV‐B3 were subsequently re‐typed as HAdV‐B114 (see below) either by complete genomic sequencing using HTS or by additional Sanger sequencing of the fiber and penton genes.

### 3.2 Complete Genomic Sequencing

Complete viral genome sequences were generated for 22 of the 67 diagnostic specimens that had initially been typed as HAdV‐B3 (imputed serotyping by partial hexon sequencing). HTS from the original samples (*n* = 6) or HAdV isolates (*n* = 16) resulted in 4.4 × 10^6^ (median, range 1.4 × 10^6^–8.3 × 10^6^) sequence reads per sample with an average sequence read coverage of 1153 (median, range 19–19 660) per sample; please see Supporting Information S1: Table S1 for more details. Sequenced specimens originated from all over Germany: five from patients from Tuebingen (GenBank #OR853835, selected as representative HAdV‐B114 prototype sequence, #PQ189738, #PQ189747, #PQ189748 (variant 1), #PQ189749), five from Hannover (#PQ189753, #PQ189754, #PQ189752, #PQ189755, #PQ189756), four from Ulm (#PQ189741, #PQ189742, #PQ189743, #PQ189744), two from Munich (#PQ189740, #PQ189746), and single samples from Bonn (#PQ189751), Heidelberg (#PQ189739), Landsberg (#PQ189737), Oberhausen (#PQ189736), Regensburg (#PQ189745), and Stuttgart (#PQ189750, variant 2). All these sequences were almost identical to the HAdV‐B114 prototype sequence with the highest pairwise distance (0.004) found in variant 2 (#PQ189750).

### 3.3 Phylogenetic Analysis Revealed the Novel Recombinant HAdV Type B114

**Figure 2:**
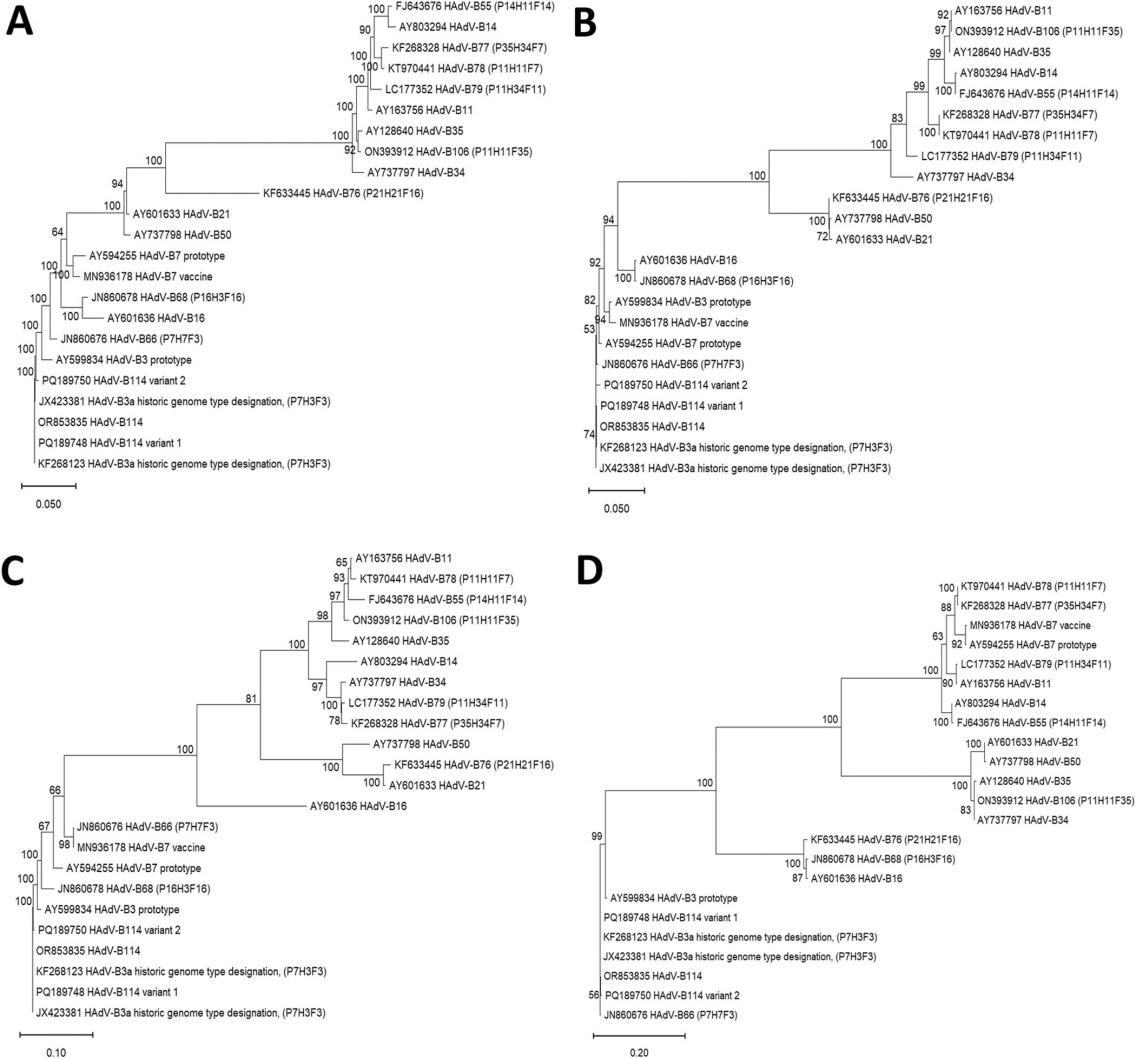
Phylogenetic analysis of the newly identified HAdV‐B114 (P7H3F3). Phylogenetic trees (maximum likelihood algorithm using RAxML) were constructed for (A) the complete adenoviral genomic sequences, (B) the penton base genes, (C) the hexon genes, and (D) the fiber genes. Bootstrap values are indicated at the nodes. Please note that sequences labeled as B3a refer to the historic genome type taxonomy.

**Figure 3:**
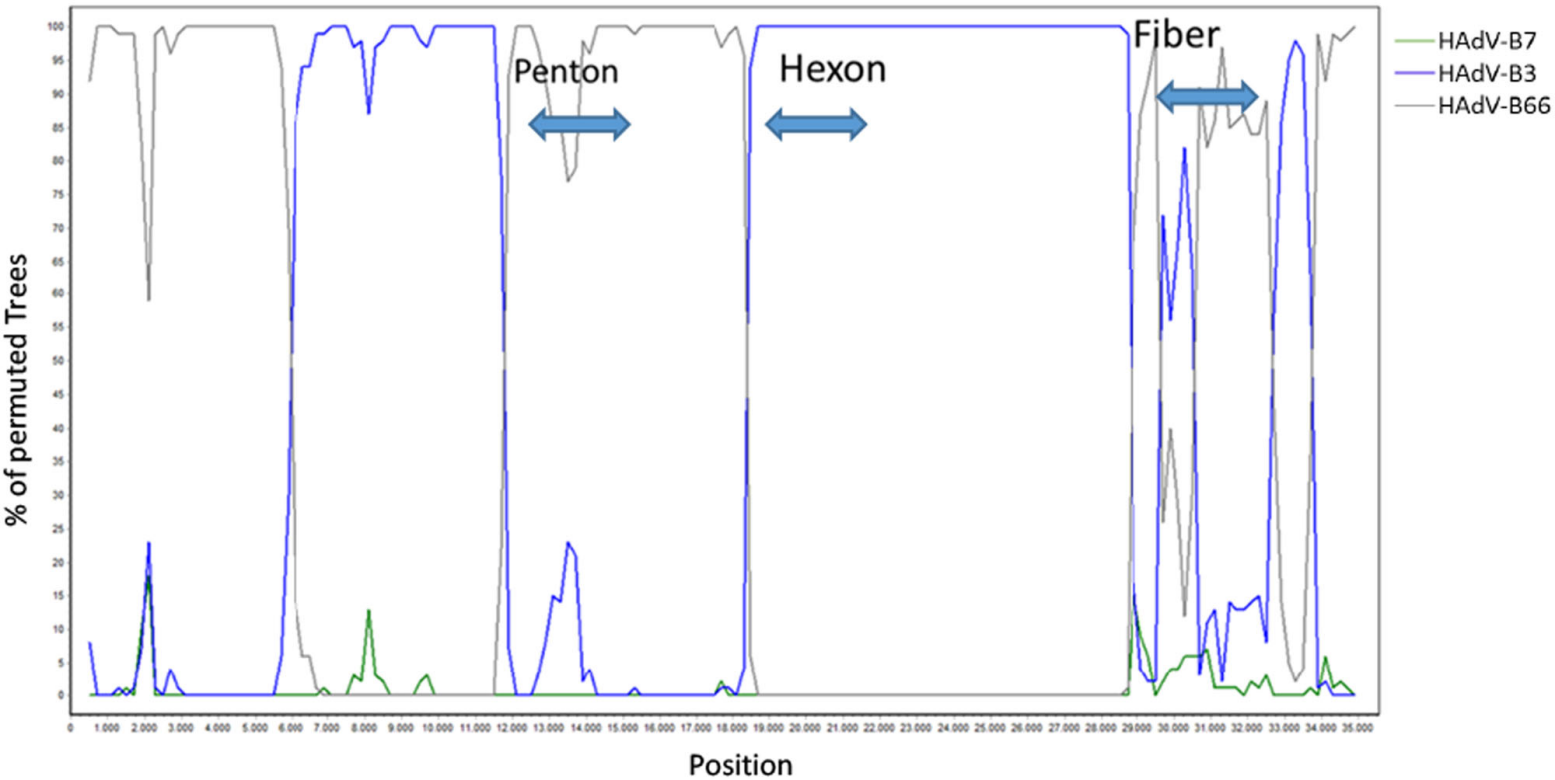
Bootscan analysis of the complete genomes of the putative HAdV‐B3 (i.e., HAdV‐B114) isolate revealed its recombinant phylogeny (P7H3F3). The penton gene region of the novel HAdV‐B114 originates from HAdV‐B7 (green colored line). Bootscan indicated HAdV‐B66 (gray colored line), but HAdV‐B66 is itself a recombinant and has a penton gene derived from HAdV‐B7. The hexon gene derives from HAdV‐B3 (blue colored line). The bootscan is not conclusive in the fiber gene region, because HAdV‐B3 and ‐B66 share an almost identical fiber sequence.

Phylogenetic trees of complete genomic sequences, as well as of the hexon, fiber, and penton genes (Figure 2) depicted the clustering of the “2023 epidemic HAdV‐B3” sequences with the HAdV‐B3 prototype in the hexon and fiber genes, but in the penton gene with the HAdV‐B7 prototype (most closely related serotype) and HAdV‐B66 (most closely related genotype). Clustering of the HAdV‐B114 penton sequence with the HAdV‐B7 prototype and HAdV‐B66 was confirmed by constructing and bootstrap testing of UPGMA, Neighbor‐Joining, Minimum Evolution, and Maximum Parsimony trees (data not shown) in addition to the Maximum Likelihood tree presented in Figure 2B. Nucleic acid identities of the penton gene between the HAdV‐B114 and the HAdV‐B7 and HAdV‐B66 prototype sequences were 1625/1635 (99.39%) and 1630/1635 (99.69%), respectively, but the HAdV‐B114 sequence compared to the HAdV‐B3 prototype sequence had only 1611/1635 (98.53%) identities. Bootscan analysis confirmed the observed recombinant phylogeny (Figure 3). Both the bootscan and the penton trees rather indicated a recombination with HAdV‐B66 than with the HAdV‐B7 prototype, but HAdV‐B66 itself is a recombinant genotype (P7H7F3) owing a penton gene derived from HAdV‐B7. Consequently, the recent HAdV‐B3 isolates were designated as the novel recombinant genotype HAdV‐B114 (P7H3F3) by the Human Adenovirus Working Group (http://hadvwg.gmu.edu/). We also analyzed in detail the phylogeny of the early gene regions E1–E4, which encode nonstructural genes. With the exception of the E2 genes, HAdV‐B114 sequences were also most closely related to HAdV‐B66 (Supporting Information S1: Figure S1).

### 3.4 Identification of Previous HAdV‐B114 Isolates (Prior to 2023) and Identity to Genome Type 3a

An initial NCBI Blast analysis of the HAdV‐B114 prototype sequence #OR853835 revealed 103 complete genomic sequences from the last two decades with > 99.8% identity in GenBank (Supporting Information S2: Supplementary Document 1). Most of these originated from clinical isolates from the USA, China, and Japan and had been labeled as HAdV‐B3, according to the then valid taxonomy. All these sequences clustered with HAdV‐B7 in the penton gene (see highlighted hits in Supporting Information S3: Supplementary Document 2) and can therefore be considered as HAdV‐B114 sequences, although a few were erroneously labeled as P3H3F3 instead of P7H3F3. This shows that HAdV‐B114 circulated in China, Japan, and the USA prior to 2023.

GenBank sequence #JX423381.1 (99.91% identity to the HAdV‐B114 prototype sequence #OR853835) had a more detailed description as variant (genome type) 3a and eight other GenBank sequences had “3a” in their strain designation, although a more detailed description was missing (#KF268123.1, #KF268133.1, #KF268120.1, #JX423380.1, #JX423381.1, #JX423382.1, #OR753121.1, #KF268133.1). This suggested that HAdV‐B114 is identical to the previously described genome type 3a. As genome types (not to be confused with genotypes) had been defined by band patterns in RFLP analysis, we performed in silico RFLP genome typing for the HAdV‐B114 prototype sequence (#OR853835), which confirmed a band pattern almost identical to genome type 3a (Supporting Information S1: Figure S2).

As no complete genomic sequence data of German HAdV‐B3 isolates prior to 2023 were available in GenBank, we (partially) sequenced the penton gene region from archived DNA extracts of diagnostic samples which had previously been typed as HAdV‐B3 by imputed serotyping. Archived DNA from 31 of 48 diagnostic samples from 2007 to 2021 was still available for penton gene sequencing. All of these samples had a hexon sequence clustering with HAdV‐B3 and a penton sequence clustering with HAdV‐B7. Thus, it can be considered that they should be re‐typed as HAdV‐B114.

### 3.5 Amino Acid Substitutions in the Penton Base Gene Compared to the HAdV‐B3 Prototype

**Figure 4:**
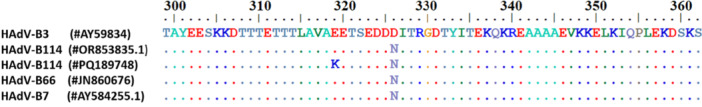
Multiple alignment of deduced amino acids of the “RGD” loop of the penton base. The sequences of the respective prototypes and the HAdV‐B114 variant 1 sequence (#PQ189748) were analyzed.

The deduced HAdV‐B114 amino acid sequence of the RGD loop in the penton base revealed a complete identity with both the HAdV‐B7 and HAdV‐B66 sequences but not with HAdV‐B3 (Figure 4). This loop of the penton base binds with its RGD sequence motif to the secondary cellular receptors, integrins, and thus the sequence of the RGD loop may influence the affinity and tropism to different integrins [7]. Of note, very close to the RGD motif at positions 329–331, HAdV‐B3 has a negatively charged glutamate (D) at position 326, whereas the positively charged asparagine (N) is found at this position in HAdV‐B114, ‐B66, and ‐B7. In addition to this crucial amino acid substitution, a multiple alignment of the complete penton base prototype sequences of HAdV‐B114, HAdV‐B3, HAdV‐B7, and HAdV‐B66 (deduced amino acid sequences) revealed only a few other substitutions. Both HAdV‐B114 and HAdV‐B66 had a common I32L substitution compared to HAdV‐B7 and HAdV‐B3, which corresponded well to the phylogenetic analysis on the nucleic acid sequence level (see above). All HAdV‐B114 sequences (except PQ189750) featured a unique A462T substitution compared to the sequences of HAdV‐B3, HAdV‐B7, and HAdV‐B66, but this substitution is distant from the RGD motif. Furthermore, a subset of HAdV‐B114 sequences (#PQ189748, #PQ189753, #PQ189752, #PQ189755, #PQ189741) had a unique E319K substitution.

### 3.6 Clinical and Virological Characteristics of Patients With Severe Disease Associated With HAdV‐B114

At the University Hospital Tübingen, three patients with systemic HAdV‐B114 disease had been treated in spring 2023: two with rather severe LRTI and one case of adenovirus‐associated thrombocytopenia with cerebral venous sinus thrombosis (clinical details summarized in Table 1). Both pneumonia patients were immunocompromised due to allogeneic hematopoietic stem cell transplantation. Although not requiring mechanical ventilation, they presented with fever, dyspnea, and high viral loads in the lower airways (Ct‐value 20–24 upon HAdV real‐time PCR) and significant DNAemia (5.2 × 10^3^ and 7.6 × 10^4^ copies/mL plasma, respectively), typical for a disseminated adenoviral disease. Both shed HAdV into the feces and presented with leukopenia. One as well had elevated liver enzymes, potentially indicating an adenoviral hepatitis. Noteworthy, the third case of severe systemic HAdV‐B114 disease presented as adenovirus‐associated thrombocytopenia and thrombotic events in a 7‐year‐old child. This case has recently been reported in detail by others [20]; however, without typing and recognizing its association to HAdV‐114.

**Table 1:** Clinical and virological characteristics of three patients with either HAdV‐B114‐associated severe lower respiratory tract infection (LRTI) or thrombosis and thrombocytopenia syndrome (TTS).

ID	TUE‐LRTI‐01	TUE‐LRTI‐02	TUE‐TTS
**Date**	Feb/Mar 23	Mar/Apr 23	Jun 23
**Age**	39	70	7
**Sex**	Male	Female	Female
**Comorbidities**	− Allogeneic stem cell transplantation	− Allogeneic stem cell transplantation	None
**Symptoms**	− Cough, sore throat − Fever − Dyspnea	− Cough, sore throat − Fever − Dyspnea, increasing respiratory insufficiency − Arterial hypotension − Emesis	− Conjunctivitis − Fever − Exanthema in the face − Arterial hypertension − Emesis − Headache − Somnolence
	− Pneumonia − HAdV‐DNAemia − Hepatitis? (elevated ALAT) − Pancytopenia	− Pneumonia − HAdV‐DNAemia − Hepatitis? (elevated ALAT) − Lymphocytopenia	− Thrombocytopenia − Cerebral sinus venous thrombosis
**Hospitalization**	8 days	8 days	32 days
**ICU stay**	None	None	20 days
**Mechanical ventilation**	None	None	8 days (orotracheal) due to high intracranial pressure
**Outcome**	Survived	Survived	Survived
**Antiviral treatment**	− Cidofovir − Aciclovir	− Aciclovir	− Aciclovir
**HAdV load peak values (copies/mL) or detection** [**Ct‐value**]	− Throat swab [Ct 17.0] − BAL [Ct 20.6] − Plasma (7.6 × 10^4^) − Stool (> 1.0 × 10^7^)	− BAL [Ct 24.0] − Plasma (5.2 × 10^3^) − Stool (6.0 × 10^2^)	− Throat swab [Ct 29.0]

*Note:* Viral loads (copies/mL or Ct‐value) were assessed using the Adenovirus R‐gene (Biomerieux) or the Panther Fusion AdV/hMPV/RV Assay (Hologic) real‐time PCR assays for plasma and stool samples or respiratory specimens, respectively. The case of HAdV‐B114‐associated thrombocytopenia and thrombotic events in a 7‐year‐old child has recently been reported in detail by others [20]; however, the recombinant phylogeny of HAdV‐114 had not been recognized at that time, therefore, it was included in this table.

Abbreviations: ALAT, alanine aminotransferase; BAL, bronchoalveolar lavage; ICU, intensive care unit.

### 3.7 Characteristics of Patients With HAdV‐B114‐Associated Eye Infections and Accompanying URTI

Supporting Information S1: Table S2 shows the clinical data of 16 patients with conjunctivitis who were treated in the ophthalmologic out‐patient clinic of the University Hospital Tuebingen between February 2023 and June 2023. With one exception (suspected mild keratitis), no involvement of the corneal structure was observed by the treating ophthalmologists. The most frequently reported symptoms were conjunctival redness, foreign body sensation, pruritus, and mild ocular pain. Three patients reported concurrent symptoms of URTI or fever, although this information was not routinely assessed and may be underestimated. It is suspected that these patients suffered from PCF. Noteworthy, 9 of 16 patients reported a recent history of similar cases of conjunctivitis and/or common cold with eye involvement in their household. We detected high HAdV loads in the conjunctival swabs by PCR (2.4 × 10^5^ to up to > 1.0 × 10^7^ copies HAdV‐DNA/mL). HAdV‐B114 infection was confirmed by genotyping (sequencing of penton, hexon, and fiber genes), and in three cases, the complete HAdV genome was sequenced. In summary, these data indicate an epidemic of PCF‐like illness with relatively frequent intrafamilial spread in Southern Germany at that time.

## 4 Discussion

Increased circulation of adenovirus was observed worldwide in 2022 and 2023 [21, 22, 23, 24, 25]. Noteworthy, the majority of respiratory adenovirus infections during this time were published to be caused by HAdV‐B3 [21, 26, 27], although the vast majority of these were probably HAdV‐B114 infections according to the published penton sequences (Supporting Information S3: Supplementary Document 2).

Previous SARS‐CoV‐2 pandemic measures (e.g., the closing down of kindergartens) also limited transmission of endemic adenoviruses and had probably resulted in an exceptionally large group of immune‐naïve infants. Similar to other viruses [28], the postpandemic reemergence of adenoviruses, such as epidemics of HAdV‐F41, and other HAdV types (e.g., HAdV‐B114), was highly likely and has been indeed observed [21, 22, 23, 24, 29]. Adenoviruses may then have spread to older immune‐naïve or otherwise highly susceptible patients, for example, immunosuppressed persons. In addition, increased awareness and behavioral changes in the general population after the pandemic may have led to increased rates of diagnosis. Although precise epidemiologic data on respiratory adenoviruses in Germany are lacking, as these are non‐notifiable, the increase in initial HAdV‐B3 typing results (later retyped as HAdV‐B114) was impressive (Figure 1).

To a lesser extent, increased numbers of the closely related type HAdV‐B7 were also detected in 2023 (Figure 1). Despite early reports on severe LRTI cases and eye infections caused by HAdV‐B3 in the 1950s–1970s [30, 31, 32, 33], HAdV‐B7 was considered to be associated with more severe LRTI and outbreaks in military barracks, and was therefore included in the only available adenovirus vaccine, developed in the 1970s, whereas HAdV‐B3 was not [34, 35]. Subsequently, genomic subtyping of clinically or epidemiologically relevant HAdV‐B7 and ‐B3 isolates by RFLP was introduced and more frequently applied to HAdV‐B7 due to its superior clinical significance [36, 37, 38, 39]. For example, genome types 7a–7g were identified in isolates originating from 1958 to 1981 in addition to the prototype (7p) originating from 1954 [36]. Genome type 7h was identified for the first time in 1984 and was associated with severe and even fatal LRTIs in South America [13, 14, 40]. By 1990, 7h had completely replaced the previously predominant genome type 7c (and other genome types) in South America [14]. Both its association with severe disease and the strain displacement in the late 1980s suggested that 7h was a novel, emerging strain of HAdV‐B7. Subsequent sequence analysis of genome type 7h revealed its recombinant molecular phylogeny with its fiber sequence deriving from HAdV‐B3 [41]. Therefore, HAdV‐B7 genome type 7h was relabeled as the recombinant genotype HAdV‐B66 (P7H7F3) in 2012, 28 years after its first isolation. The penton base sequence of HAdV‐B66 was found to be slightly evolved, but still was considered as type 7‐like [1].

In case of the recent epidemic of what was initially considered to be caused by a strain of HAdV‐B3, analysis of the isolates was performed by complete genomic sequencing, and retyping as genotype HAdV‐B114 (P7H3F3) was achieved within less than a year. However, initial NCBI Blast analysis of these complete genomic sequences gave somewhat misleading results, showing a very high sequence identity (> 99.8%) to a multitude of complete genome sequences from the USA and China labeled as HAdV‐B3 (some of these even erroneously labeled as P3H3F3) which have to be considered as HAdV‐B114 (P7H3F3) (Supporting Information S2 and S3: Supplementary Documents 1 and 2). Mislabeling of genotypes in the databases is a general problem that is not easy to mitigate. Common causes for annotation errors in public databases are contamination of biological samples, incorrect metadata submission by users, or errors related to bioinformatic/computational tools depending on applied settings [42]. In case of the sequences erroneously labeled as P3H3F3 instead of P7H3F3, errors related to the analysis of their recombinant phylogeny seem to be probable. Although most of the genomic sequences were recent clinical isolates originating from China and the USA suggesting significant HAdV‐B114 circulation in these countries, a few were labeled as the old genome type 3a: #KF268123 with 99.97% identity and #JX423381.1 with 99.91% identity to HAdV‐B114 (for comparison, the identity of the HAdV‐B114 sequence to the HAdV‐B3 prototype sequence #AY599834.1 was only 98.44%). Since genome type 3a was originally defined by its RFLP patterns [37], we performed in silico RFLP testing on our HAdV‐B114 sequence data, which confirmed its identity with the genome type HAdV‐B3a (Supporting Information S1: Figure S2). Therefore, HAdV‐B114 was not considered to be a novel, emerging virus (like HAdV‐B66 in the 1980s), but rather a reemergent virus, previously labeled as genome type 3a.

According to the usual taxonomy procedures for genome types, the HAdV‐B3 prototype from 1953 was labeled as genome type 3p [37, 38] and the oldest HAdV‐B3 strains with a different RFLP pattern isolated as early as 1962 in Japan, China, and the USA (but not in Europe and South America) were classified as genome type 3a [38, 43]. Very closely related strains were labeled as 3a1–3a7, and subsequent strains discerned by RFLP were labeled as 3b, 3c, and so on [37, 38, 43, 44]. Interestingly, genome type 3a was associated with rather severe disease compared to other HAdV‐B3 genome types. In addition to URTI, its clinical manifestations included LRTI, eye infections, and even a few disseminated and central nervous system infections [37, 38, 43]. These clinical presentations of HAdV‐B3a infections appear to be identical to the diseases caused by HAdV‐B114 in 2023. As genomic sequencing was not yet feasible when HAdV‐B3a was isolated, the emergence of the more virulent genome type 3a was then considered to be a result of positive selection of point mutations rather than a result of recombinant phylogeny and thus HAdV‐B114 was not discovered until 2023 [37].

Although the hexon contains the main neutralization determinant, fiber and penton base proteins bind to primary and secondary cellular receptors and therefore rather influence tropism and virulence. It is intriguing that HAdV‐B114 (previously HAdV‐B3a) and HAdV‐B66 (previously HAdV‐B7h) share almost identical penton and fiber base sequences (P7 and F3) and both HAdV‐B3a and ‐B7h were considered to be more virulent than their corresponding prototypes [13, 14, 37, 38, 40, 43]. Moreover, HAdV‐B114 and HAdV‐B66 were closely related in several early genes, which may influence virulence and pathogenicity (Supporting Information S1: Figure S1). Furthermore, if we assume that recent reports on HAdV‐B3 cases represent unrecognized HAdV‐B114 infections, our results on circulation and clinical severity are in line with recent studies on an increased circulation of HAdV in the USA in 2023, with 71.9% being typed as HAdV‐B3 (based on HAdV hexon sequencing, thus a differentiation of HAdV‐B3 and ‐B114 was not possible) [21]. These HAdV‐B3‐infected patients were frequently found in the age group between 3 and 5 years [21], but no increased rates of hospitalization were associated with HAdV‐B3 infections. Older age and immunosuppression were the top two variables associated with an increased likelihood of hospital admissions with HAdV in the latter study, similar to our observations.

The limitations of this study are the following: first, our study does not indicate that HAdV‐B114 evolved by a single recombination event that replaced the penton gene of HAdV‐B3 with an HAdV‐B7 penton sequence. This may be erroneously suggested by the taxonomic labeling as P7H3F3. HAdV‐B114 probably evolved through several recombination events involving HAdV‐B66 (with a genomic backbone evolved from HAdV‐B7) and HAdV‐B3 as suggested by the phylogenetic trees of its early and late gene regions as well as by bootscan analysis. Possible further steps that could be taken in the future include conducting a phylogenetic analysis to determine the possible order of events (e.g., molecular clock or detailed recombination breakpoint analyses). Second, although biological experiments to investigate the potentially enhanced transmission or pathogenicity of HAdV‐B114 could be very useful, they are beyond the scope of this study.

In conclusion, retrospective NCBI BLAST analysis, penton base gene sequencing of older German HAdV‐B3 isolates and comparison of RFLP patterns to results of studies from the 1980s unequivocally indicate that HAdV‐B114 is not a novel emerging virus, but has been circulating already for decades. Nevertheless, the reemergence of HAdV‐B114, which can be considered to be more pathogenic than the closely related HAdV‐B3 prototype, caused a significant disease burden, which eventually led to its identification as a recombinant HAdV genotype.

## Author Contributions

Conceptualization: Tina Ganzenmueller, Albert Heim. Methodology: Lars Steinbrück, Albert Heim. Resources: Magnus Wolf, Charikleia Gkioule, Lasse Wolfram, Lars Steinbrück, Albert Heim. Formal analysis: Tina Ganzenmueller, Magnus Wolf, Charikleia Gkioule, Lars Steinbrück, Albert Heim. Validation: Albert Heim. Software: Lars Steinbrück. Writing – original draft: Tina Ganzenmueller, Magnus Wolf, Albert Heim. Writing – review and editing: Albert Heim, Magnus Wolf, Lasse Wolfram, Tina Ganzenmueller. All authors read and approved the final version of the manuscript.

## Ethics Statement

This study (analysis of clinical data of pseudonymized patients together with viral sequencing data) was approved by the Ethics Committee of the University Hospital Tuebingen and individual patient consent was waived (No. 774/2023BO2).

## Conflicts of Interest

The authors declare no conflicts of interest.

## Supporting information

Supplementary Data Supp Methods Table S1 Table S2 Fig S1 Fig 2 revised final.

Supplementary Document 1 BLAST Complete Genome revised.

Supplementary Document 2 BLAST Penton revised.

## Data Availability

The GenBank accession number for the novel HAdV‐B114 whole genome sequence is OR853835 (prototype sequence). Additional genomic sequences generated for this study have been deposited under accession numbers PQ189736–PQ189756.
