# Supplementary material for: An Epidemic of Respiratory and Ocular Infections Caused by the Reemergence of a Recombinant Human Adenovirus, the Novel Type HAdV‐B114 (P7H3F3)
Source: J Med Virol. 2025 Jun 30;97(7):e70464. doi: 10.1002/jmv.70464 (PMC12208009; doi:10.1002/jmv.70464)
Supplement: Supplementary file 1 — Supplementary Data Supp Methods Table S1 Table S2 Fig S1 Fig 2 revised final. [file JMV-97-e70464-s002.docx]

**Supplementary Data**

**Supplementary Information on Methods**

**Library construction and sequencing**

Between 3ng and 280ng total DNA per sample was used as input for the NEB Ultra II FS Library Preparation Kit. After fragmentation (7min 30s) according to the standard protocol and subsequent size selection (aiming for a mean insert size of around 500bp), libraries were indexed using 8bp Dual-Unique-Indices (NEB), pooled and loaded at a final concentration of 10 to 12pM onto a MiSeq using v2/v3 sequencing kits. The number of PCR cycles was adjusted according to the input concentration as recommended by the manufacturer.

**Genome assembly and annotation**

Sequencing reads were trimmed by using fastp 0.23.1 (-q20; -l 50) and mapped against the human genome (hg19), using bowtie2 2.3.5.1. The remaining, unaligned reads were extracted using samtools or SamToFastq (Picard) and used for *de novo* assembly. Assembly was performed by using SPAdes 3.15.4 (--isolate), Minia3 (kmer-size = ½ mean insert size), GATB (kmer {121,161,201,221,241}; no scaffolding) and MEGAHIT. After combining the resulting contigs and removing all sequences <500 nucleotides, a scaffolding step was performed by mapping the contigs to reference sequence with Minimap2 2.17-r941 (-x asm20) and pulling the consensus with samtools mpileup 1.10 (-d 500, -A, --ff UNMAP –no-BAQ, -q 0) and iVar 1.3.1 (-q 0, -t 0, -c 0.8, -m 1). Subsequently the draft genome underwent correction steps: raw reads were strictly trimmed with fastp (--cut_right; --cut_right_mean_quality 20; -l 30), used for gap filling with abyss-sealer 2.3.7 (-B64G, -k{33,64,80,96,112,128}, -g 1000), followed by Gap2Seq (--dist-error 1000, -k96)+Gap2Seq with read filtration (--dist-error 1000, bam-file/mean-insert-size/30/40). Finally, these strictly trimmed reads were used for polishing (NextPolish 1.4.1; rerun = 3) and afterwards mapped back to the gap-filled, polished draft genome, using bowtie2 and polished with pilon 1.24 (--fix-all, --no-strays). This step was repeated twice. As a final correction step, we used a GATK4 (4.2.2.0-1) pipeline following “best practices” of GATK. Reads from the Pilon correction step were trimmed again with fastp (--cut_right; --cut_right_mean_quality 30; -l 30), mapped to the Pilon-corrected genome and variant calling was performed, using bcftools 1.4 ‘mpileup’, ‘call’ and ‘filter’ ('%QUAL<20 || DP<10'), to create a “known variants” input table for the GATK pipeline. After deduplication of the reads with PICARD 2.25.1, the mapping files underwent a BaseQualityScoreRecalibration (BQSR) and the Haplotype-caller (-ploidy 1, -stand-call-conf 30.0, --adaptive-pruning true) was used to call the variants. After filtering and merging of the resulting tables, all variants with a frequency of >50% were replaced in the consensus sequence by FastaAlternateReferenceMaker (GATK). Results were checked and annotations were transferred using Geneious Prime (90% similarity cut-off) from a collection of HAdV reference sequences, using the match with the highest similarity.

**Supplementary Table S1. Result data of adenovirus genome sequencing by high-throughput sequencing (HTS).** Basic sequencing results achived by Illumina sequencing (Miseq) in the HAdV-positive samples (n=6 diganostic samples and n=18 virus isolates) are displayed. Please note that the HAdV load (copies per ml original diagnostic specimen, as determined by real-time PCR) were only available if we sequenced directly from the diagnostic specimen. The adenoviral cell culture isolates usually contain high virus titers, which is also resembled by the excellent sequencing depth in these samples

| **GenBank Name** | **Accession number** | **Seq. depth**  **(mean read coverage)** | **Total reads**  **(n)** | **Host content (%)** | **Sampling date** | **sequenced material & viral load, (copies/ml)** |
| --- | --- | --- | --- | --- | --- | --- |
| 43593/Han/1/2023 | **OR853835** | 101 | 3.536.396 | 94.74 | 04/2023 | cell culture isolate |
| 24274/Han/2/2023 | **PQ189736** | 1576 | 3.718.794 | 83.78 | 02/2023 | cell culture isolate |
| 25355/Han/3/2023 | **PQ189737** | 774 | 5.057.928 | 89.61 | 02/2023 | cell culture isolate |
| 36437/Han/4/2023 | **PQ189738** | 35 | 4.876.602 | 94.23 | 03/2023 | original sample  (>1.0 x 10^8^  copies/ml) |
| 38730/Han/6/2023 | **PQ189739** | 314 | 3.374.868 | 91.30 | 03/2023 | original sample  (5.4 x 10^7^ copies/ml) |
| 51980/Han/9/2023 | **PQ189740** | 3313 | 4.157.222 | 78.30 | 05/2023 | cell culture isolate |
| 55149/Han/10/2023 | **PQ189741** | 11967 | 8.828.098 | 64.98 | 05/2023 | cell culture isolate |
| 55156/Han/11/2023 | **PQ189742** | 19660 | 7.349.772 | 31.99 | 05/2023 | cell culture isolate |
| 55158/Han/12/2023 | **PQ189743** | 1531 | 8.182.228 | 90.34 | 05/2023 | cell culture isolate |
| 55162/Han/13/2023 | **PQ189744** | 7413 | 7.162.872 | 70.03 | 05/2023 | cell culture isolate |
| 63735/Han/14/2023 | **PQ189745** | 53 | 1.573.608 | 68.80 | 06/2023 | cell culture isolate |
| 66048/Han/15/2023 | **PQ189746** | 3048 | 1.450.350 | 46.86 | 06/2023 | cell culture isolate |
| 66165/Han/16/2023 | **PQ189747** | 198 | 1.881.478 | 92.55 | 03/2023 | original sample  (>1.0 x 10^8^ copies/ml) |
| 66198/Han/17/2023 | **PQ189748** | 19 | 1.352.432 | 96.04 | 06/2023 | original sample  (>1.0 x 10^8^ copies/ml) |
| 66200/Han/18/2023 | **PQ189749** | 337 | 1.656.272 | 91.99 | 06/2023 | original sample  (>1.0 x 10^8^ copies/ml) |
| 73731/Han/20/2023 | **PQ189750** | 3521 | 5.742.194 | 82.50 | 07/2023 | cell culture isolate |
| 74412/Han/21/2023 | **PQ189751** | 179 | 7.249.522 | 95.12 | 07/2023 | original sample  (>1.0 x 10^8^ copies/ml) |
| 30179/Han/8/2023 | **PQ189752** | 8616 | 4.945.280 | 56.74 | 02/2023 | cell culture isolate |
| 36846/Han/5/2023 | **PQ189753** | 97 | 3.510.332 | 96.19 | 03/2023 | cell culture isolate |
| 48508/Han/7/2023 | **PQ189754** | 10618 | 4.594.658 | 37.61 | 04/2023 | cell culture isolate |
| 58834/Han/19/2023 | **PQ189755** | 187 | 1.478.436 | 91.37 | 05/2023 | cell culture isolate |
| 71573/Han/22/2023 | **PQ189756** | 13236 | 6.472.694 | 45.43 | 07/2023 | cell culture isolate |

**Supplementary Table S2. HAdV-B114-associated conjunctivitis.** Clinical (i.e. symptoms) and epidemiologic (i.e. relation to other cases) characteristics and detected HAdV loads (copies/ml swab medium) in the respective eye swabs are shown for the Tuebingen conjunctivitis cases.

| **ID** | **Date** | **Age [years]** | **Sex** | **Disease** | **Similar case in the household?** | **Viral load from conjunctival swab** |
| --- | --- | --- | --- | --- | --- | --- |
| **TUE-CONJ-01** | Feb 23 | 39 | male | *conjunctivitis* |  | 2.0 x 10^7^ c/ml; *Ct* 19.8 |
| **TUE-CONJ-02** | Feb 23 | 6 | male | *conjunctivitis* |  | 2.4 x 10^5^ c/ml; *Ct* 27.9 |
| **TUE-CONJ-03** | Mar 23 | 34 | male | *conjunctivitis* |  | > 1.0 x 10^8^ c/ml; *Ct* 16.9 |
| **TUE-CONJ-04** | Mar 23 | 33 | male | *conjunctivitis* | yes (parent) | > 1.0 x 10^8^ c/ml; *Ct* 13.0 |
| **TUE-CONJ-05** | Apr 23 | 42 | male | *conjunctivitis* | yes (parent) | > 1.0 x 10^8^ c/ml; *Ct* 15.0 |
| **TUE-CONJ-06** | Apr 23 | 37 | female | *conjunctivitis* |  | 2.1 x 10^7^ c/ml; *Ct* 18.4 |
| **TUE-CONJ-07** | May 23 | 39 | female | *conjunctivitis* / *nasopharyngitis* |  | 9.4 x 10^5^ c/ml; *Ct* 24.5 |
| **TUE-CONJ-08** | May 23 | 52 | male | *conjunctivitis* | yes (children) | 2.4 x 10^6^ c/ml; *Ct* 22.4 |
| **TUE-CONJ-09** | Jun 23 | 40 | male | *conjunctivitis* | yes (child) | 8.4 x 10^7^ c/ml; *Ct* 17.4 |
| **TUE-CONJ-10** | Jun 23 | 33 | female | *conjunctivitis* / sinusitis | yes (children) | 4.4 x 10^5^ c/ml; *Ct* 24.2 |
| **TUE-CONJ-11** | Jun 23 | 10 | male | *conjunctivitis* / *nasopharyngitis* |  | 4.9 x 10^7^ c/ml; *Ct* 19.0 |
| **TUE-CONJ-12** | Jun 23 | 0 | female | *conjunctivitis* / acute febrile infection | yes (parent = TUE-CONJ-13) | 7.1 x 10^6^ c/ml; *Ct* 21.3 |
| **TUE-CONJ-13** | Jun 23 | 32 | female | *conjunctivitis* | yes (child = TUE-CONJ-12) | 4.1 x 10^7^ c/ml; *Ct* 18.7 |
| **TUE-CONJ-14** | Jun 23 | 48 | male | *keratoconjunctivitis* | yes (spouse = TUE-CONJ-15) | 6.4 x 10^7^ c/ml; *Ct* 17.8 |
| **TUE-CONJ-15** | Jun 23 | 47 | female | *conjunctivitis* | yes (spouse = TUE-CONJ-14) | > 1.0 x 10^8^ c/ml; *Ct* 14.6 |
| **TUE-CONJ-16** | Jun 23 | 35 | female | *conjunctivitis* | none | > 1.0 x 10^8^ c/ml; *Ct* 11.0 |

**Suppl. Fig 1.**

**Supp Fig, S1 (cont.)**

**Suppl. Fig 1.** Phylogenetic trees (maximum likelihood algorithm using RAxML) were constructed for **(A)**  E1A, **(B)** E1B, **(C) E2A,** **(D)** E2B, **(E)** E3. **(F)** E4. Bootstrap values are indicated at the nodes. Please note that sequences labelled as B3a refer to the historic genome type taxonomy.**Supplementary Figure 2**

**
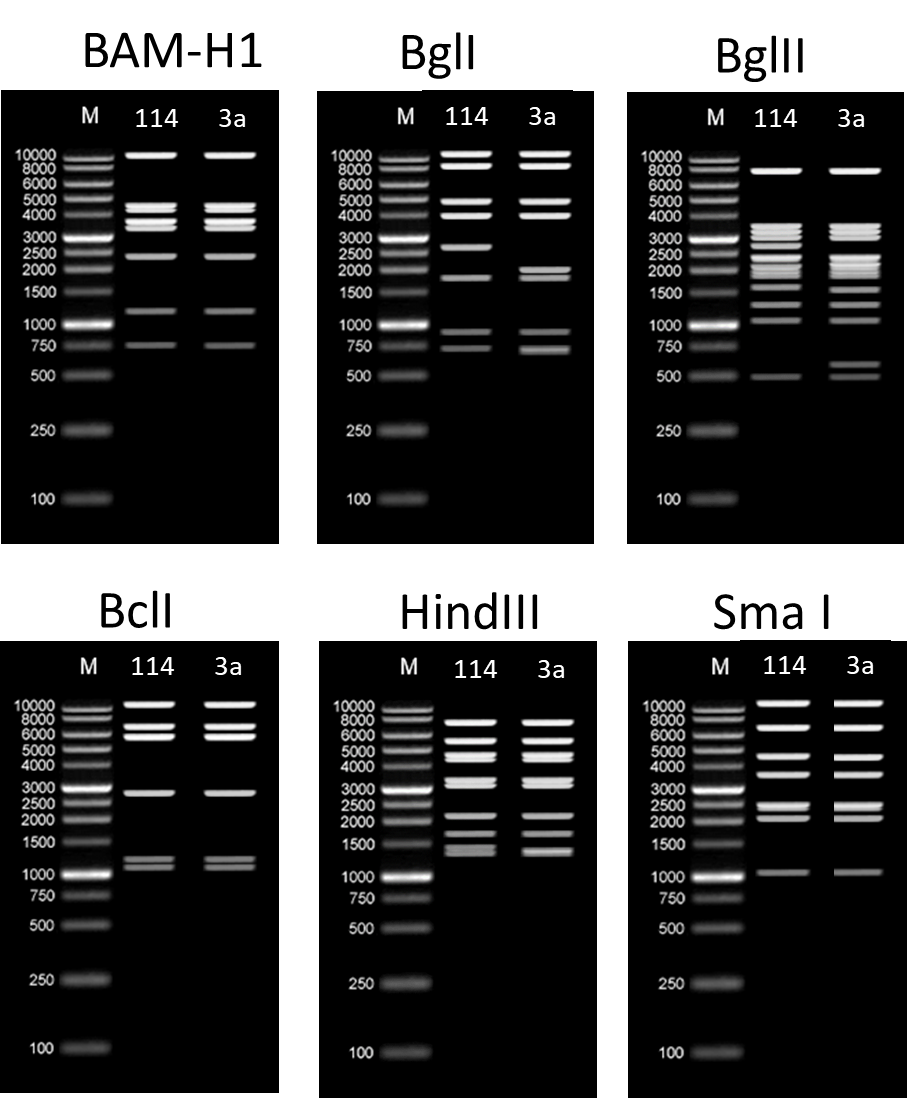
**

**Suppl. Fig 2. *In silico* RFLP testing for HAdV-B114 confirmed its identity with the genome type HAdV-B3a.** The band pattern of HAdV-B114 is almost identical to genome type 3a. Virtual restriction fragment length polymorphism (RFLP) patterns for the complete genomic sequences owere generated using the online tool Restriction Analyzer ( <https://molbiotools.com/restrictionanalyzer.php> ).
