# Supplementary material for: An Epidemic of Respiratory and Ocular Infections Caused by the Reemergence of a Recombinant Human Adenovirus, the Novel Type HAdV‐B114 (P7H3F3)
Source: J Med Virol. 2025 Jun 30;97(7):e70464. doi: 10.1002/jmv.70464 (PMC12208009; doi:10.1002/jmv.70464)
Supplement: Supplementary file 2 — Supplementary Document 1 BLAST Complete Genome revised. [file JMV-97-e70464-s003.pdf]

Supplementary document 1 BLAST Analysis Complete Genome HAdV-B114

RID: DP20Y7M0016
Job Title:OR853835:Human mastadenovirus B114 isolate...
Program: BLASTN
Database: core_nt Core nucleotide BLAST database
Query #1: Human mastadenovirus B114 isolate 43593/Han/1/2023, compete genome
Query ID: gb|OR853835.1 Length:35262

Sequences producing significant alignments:

Description	Scientific Name	Common Name	Taxid	Max Score	Total Score	Query cover	E Value	Per. Ident	Acc. Len	Accession
Human mastadenovirus B114 isolate 43593/Han/1/2023, complete genome	Human mastad..	NA	3122028	65117	65504	100%	0.0	100.00	35262	OR853835.1
Human adenovirus B3 isolate HAdV-B3/USA/1B10/2020, complete genome	Human adenov..	NA	45659	65051	65441	99%	0.0	99.97	35258	OR777156.1
Human adenovirus B3 isolate BJ20180708, complete genome	Human adenov..	NA	45659	65047	65433	99%	0.0	99.97	35255	MW748657.1
Human adenovirus B3 isolate BJ20180080, complete genome	Human adenov..	NA	45659	65045	65432	99%	0.0	99.97	35253	MW748648.1
Human adenovirus B3 isolate HAdV-B3/USA/5K5/2009, complete genome	Human adenov..	NA	45659	65041	65443	100%	0.0	99.96	35272	OQ518267.1
Human adenovirus B3 isolate HAdV-B3/USA/7K5/2011, complete genome	Human adenov..	NA	45659	65040	65441	100%	0.0	99.96	35270	OQ518278.1
Human adenovirus B3 isolate HAdV-B3/USA/16M5/2019, complete genome	Human adenov..	NA	45659	65040	65430	99%	0.0	99.97	35259	OR777235.1
Human adenovirus B3 isolate HAdV-B3/USA/6J2/2010, complete genome	Human adenov..	NA	45659	65040	65441	100%	0.0	99.96	35268	OR777168.1
Human adenovirus B3 isolate HAdV-B3/USA/10N6/2013, complete genome	Human adenov..	NA	45659	65038	65424	99%	0.0	99.97	35254	OQ518317.1
Human adenovirus B3 isolate HAdV-B3/USA/10N2/2013, complete genome	Human adenov..	NA	45659	65038	65428	99%	0.0	99.96	35258	OQ518287.1
Human mastadenovirus B Nara-A230054 DNA, complete genome	Human mastad..	NA	108098	65038	65424	99%	0.0	99.97	35256	LC799989.1
Human adenovirus B strain human/USA/UFL_Adv3a51/2007/3[P3H3F3], complete genom	Human mastad..	NA	108098	65038	65424	99%	0.0	99.97	35256	KF268123.1
Human adenovirus B3 isolate HAdV-B3/USA/10Q1/2013, complete genome	Human adenov..	NA	45659	65036	65422	99%	0.0	99.97	35254	OQ518322.1
Human adenovirus B3 isolate HAdV-B3/USA/10Q2/2013, complete genome	Human adenov..	NA	45659	65036	65426	99%	0.0	99.97	35256	OQ518344.1
Human mastadenovirus B Nara-A230042 DNA, complete genome	Human mastad..	NA	108098	65036	65422	99%	0.0	99.97	35254	LC799986.1
Human adenovirus B3 isolate HAdV-B3/USA/7G3/2011, complete genome	Human adenov..	NA	45659	65034	65424	99%	0.0	99.96	35261	OQ518306.1
Human mastadenovirus B isolate Human/China/Shanghai/3096/2009/3[P3H3F3], complet	Human mastad..	NA	108098	65032	65413	99%	0.0	99.96	35256	MK836311.1
Human adenovirus B3 isolate HAdV-B3/USA/9N2/2012, complete genome	Human adenov..	NA	45659	65032	65411	99%	0.0	99.97	35249	PP068614.1
Human adenovirus B3 isolate HAdV-B3/USA/4R8/2009, complete genome	Human adenov..	NA	45659	65030	65420	99%	0.0	99.96	35260	OQ518276.1
Human mastadenovirus B isolate Human/China/Shanghai/3496/3[P3H3F3]/2009, complet	Human mastad..	NA	108098	65030	65417	99%	0.0	99.96	35255	MK847517.1
Human mastadenovirus B Kobe-230181 DNA, complete genome	Human mastad..	NA	108098	65030	65417	99%	0.0	99.96	35255	LC799980.1
Human adenovirus B3 isolate HAdV-B3/USA/5J7/2009, complete genome	Human adenov..	NA	45659	65029	65430	100%	0.0	99.96	35268	OQ518323.1
Human adenovirus B3 isolate HAdV-B3/USA/5K2/2009, complete genome	Human adenov..	NA	45659	65029	65419	99%	0.0	99.96	35262	OQ518281.1
Human adenovirus B3 isolate HAdV-B3/USA/6R5/2010, complete genome	Human adenov..	NA	45659	65029	65419	99%	0.0	99.96	35258	OR777169.1
Human mastadenovirus B Nara-A230040 DNA, complete genome	Human mastad..	NA	108098	65027	65413	99%	0.0	99.96	35256	LC799984.1
Human adenovirus B3 isolate HAdV-B3/USA/6I2/2010, complete genome	Human adenov..	NA	45659	65025	65419	99%	0.0	99.96	35262	OQ518321.1
Human adenovirus B3 isolate BJ20180075, complete genome	Human adenov..	NA	45659	65025	65411	99%	0.0	99.96	35255	MW748647.1
Human mastadenovirus B Nara-A230077 DNA, complete genome	Human mastad..	NA	108098	65025	65411	99%	0.0	99.96	35254	LC799996.1
Human adenovirus B3 isolate HAdV-B3/USA/5S10/2010, complete genome	Human adenov..	NA	45659	65025	65411	99%	0.0	99.96	35254	OQ518265.1
Human mastadenovirus B isolate Human/China/Shanghai/4010/2012/3[P3H3F3], complet	Human mastad..	NA	108098	65023	65406	99%	0.0	99.96	35254	MK883608.1
Human adenovirus B3 isolate HAdV-B3/USA/6Q2/2010, complete genome	Human adenov..	NA	45659	65023	65413	99%	0.0	99.96	35258	OQ518260.1
Human adenovirus B3 isolate ZJ20150106, complete genome	Human adenov..	NA	45659	65019	65406	99%	0.0	99.96	35255	MW748670.1
Human adenovirus B3 isolate HAdV-B3/USA/12O4/2015, complete genome	Human adenov..	NA	45659	65017	65413	100%	0.0	99.95	35271	OR777196.1
Human adenovirus B3 isolate BJ20180730, complete genome	Human adenov..	NA	45659	65017	65419	100%	0.0	99.95	35271	MW748659.1
Human adenovirus B3 isolate BJ20180705, complete genome	Human adenov..	NA	45659	65016	65402	99%	0.0	99.95	35257	MW748656.1
Human adenovirus B3 isolate ZJ20150114, complete genome	Human adenov..	NA	45659	65016	65402	99%	0.0	99.95	35256	MW748672.1
Human adenovirus B3 isolate BJ20180718, complete genome	Human adenov..	NA	45659	65016	65402	99%	0.0	99.95	35257	MW748658.1
Human adenovirus B3 isolate HAdV-B3/USA/9C1/2019, complete genome	Human adenov..	NA	45659	65014	65415	100%	0.0	99.95	35270	OR777175.1
Human adenovirus B3 isolate HAdV-B3_8530_May_2023, complete genome	Human adenov..	NA	45659	65014	65415	100%	0.0	99.95	35269	OR487155.1
Human mastadenovirus B isolate human/China/Shanghai/678/2009/3[P3H3F3], complete	Human mastad..	NA	108098	65012	65395	99%	0.0	99.95	35254	MK836308.1
Human adenovirus B3 isolate HB20140057, complete genome	Human adenov..	NA	45659	65010	65396	99%	0.0	99.95	35256	MW748664.1
Human mastadenovirus B Kobe190508 DNA, complete genome	Human mastad..	NA	108098	65010	65396	99%	0.0	99.95	35256	LC791180.1
Human adenovirus B3 isolate BJ20170284, complete genome	Human adenov..	NA	45659	65010	65387	99%	0.0	99.95	35256	MW748643.1
Human adenovirus B3 isolate BJ20170320, complete genome	Human adenov..	NA	45659	65008	65411	99%	0.0	99.95	35255	MW748645.1
Human mastadenovirus B Nara-A230068 DNA, complete genome	Human mastad..	NA	108098	65008	65395	99%	0.0	99.95	35254	LC799994.1
Human adenovirus B3 isolate BJ20180567, complete genome	Human adenov..	NA	45659	65008	65395	99%	0.0	99.95	35255	MW748651.1
Human adenovirus B3 isolate HAdV-B3/USA/12P2/2015, complete genome	Human adenov..	NA	45659	65008	65398	99%	0.0	99.95	35260	OR777198.1
Human adenovirus B3 isolate HAdV-B3/USA/11D3/2013, complete genome	Human adenov..	NA	45659	65006	65391	99%	0.0	99.95	35258	OQ518292.1
Human adenovirus B3 isolate CC20150103, complete genome	Human adenov..	NA	45659	65005	65391	99%	0.0	99.95	35257	MW748662.1
Human adenovirus B3 isolate BJ20170281, complete genome	Human adenov..	NA	45659	65003	65382	99%	0.0	99.95	35253	MW748642.1
Human adenovirus B3 isolate GZ20150036, complete genome	Human adenov..	NA	45659	65001	65387	99%	0.0	99.95	35259	MW748663.1
Human mastadenovirus B strain Human/China/Tongliao/2019/6[P3H3F3], complete genor	Human mastad..	NA	108098	64993	65380	99%	0.0	99.94	35256	MW767985.1
Human adenovirus B3 isolate BJ20180612, complete genome	Human adenov..	NA	45659	64988	65389	100%	0.0	99.93	35280	MW748653.1
Human adenovirus B3 isolate BJ20180775, complete genome	Human adenov..	NA	45659	64988	65374	99%	0.0	99.94	35262	MW748661.1
Human adenovirus B3 isolate BJ20170379, complete genome	Human adenov..	NA	45659	64984	65371	99%	0.0	99.94	35258	MW748646.1
Human mastadenovirus B strain vzhadvb1, complete genome	Human mastad..	NA	108098	64981	65367	99%	0.0	99.94	35265	MH828478.1
Human adenovirus B3 isolate BJ20180641, complete genome	Human adenov..	NA	45659	64973	65359	99%	0.0	99.93	35264	MW748654.1
Human adenovirus B3 isolate BJ20180444, complete genome	Human adenov..	NA	45659	64968	65354	99%	0.0	99.93	35264	MW748650.1
Human adenovirus B3 isolate HAdV-B3/USA/7Q1/2011, complete genome	Human adenov..	NA	45659	64960	65361	100%	0.0	99.92	35269	OQ518299.1
Human adenovirus B3 isolate BJ20180734, complete genome	Human adenov..	NA	45659	64955	65341	99%	0.0	99.92	35263	MW748660.1
Human adenovirus B3 isolate HAdV-B3/USA/2E9/2009, complete genome	Human adenov..	NA	45659	64949	65345	100%	0.0	99.91	35268	OQ518315.1
Human adenovirus B3 isolate HAdV-B3/USA/9A4/2012, complete genome	Human adenov..	NA	45659	64945	65330	99%	0.0	99.92	35258	OQ518266.1
Human mastadenovirus B isolate Human/China/Shanghai/3517/2011/3[P3H3F3], complet	Human mastad..	NA	108098	64944	65326	99%	0.0	99.92	35252	MK883603.1
Human adenovirus B3 isolate SH20160051, complete genome	Human adenov..	NA	45659	64942	65308	99%	0.0	99.92	35249	MW748666.1
Human adenovirus B3 isolate SH20160055, complete genome	Human adenov..	NA	45659	64942	65308	99%	0.0	99.92	35249	MW748667.1
Human adenovirus B strain human/USA/ak33_AdV3a/2003/3[P3H3F3], complete genome	Human mastad..	NA	108098	64936	65324	99%	0.0	99.91	35262	JX423381.1
Human adenovirus B3 isolate GZ/H201905067/2019, partial genome	Human adenov..	NA	45659	64933	65214	99%	0.0	99.96	35204	MZ540961.1
Human adenovirus B3 isolate WZ20150066, complete genome	Human adenov..	NA	45659	64929	65315	99%	0.0	99.91	35269	MW748668.1
Human adenovirus B3 isolate HAdV-B3/USA/8E3/2011, complete genome	Human adenov..	NA	45659	64929	65319	99%	0.0	99.91	35258	OR753121.1

Human adenovirus B strain human/USA/UFL_Adv3a50/2007/3[P3H3F3]...	Human	mastad..	NA	108098	64929	65313	99%	0.0	99.91	35258	KF268133.1
Human mastadenovirus B isolate P1	Human	mastad..	NA	108098	64925	65311	99%	0.0	99.91	35265	MW013769.1
Human mastadenovirus B isolate human/China/Shanghai/538/2009/3[P3H3F3], complete	Human	mastad..	NA	108098	64925	65308	99%	0.0	99.91	35251	MK813915.1
Human adenovirus B strain human/USA/UFL_Adv3a2/2007/3[P3H3F3],complete genome	Human	mastad..	NA	108098	64923	65310	99%	0.0	99.91	35259	KF268120.1
Human adenovirus B3 isolate WZ20150082, complete genome	Human	adenov..	NA	45659	64920	65302	99%	0.0	99.91	35251	MW748669.1
Human mastadenovirus B Nara-A230057 DNA, complete genome	Human	mastad..	NA	108098	64920	65201	99%	0.0	99.96	35201	LC799992.1
Human mastadenovirus B isolate human/China/Shanghai/1631/2009/3[P3H3F3], complete	Human	mastad..	NA	108098	64914	65302	99%	0.0	99.90	35255	MK836310.1
Human adenovirus B3 isolate ZJ20150111, complete genome	Human	adenov..	NA	45659	64912	65299	99%	0.0	99.90	35269	MW748671.1
Human adenovirus B strain human/CHN/Ad4/2007/NEW[P3H3F7],complete genome	Human	mastad..	NA	108098	64909	65295	99%	0.0	99.90	35265	KF268311.1
Human adenovirus B3 isolate HAdV-B3/USA/8T6/2012, complete genome	Human	adenov..	NA	45659	64909	65295	99%	0.0	99.90	35263	OR753127.1
Human adenovirus B3 isolate HAdV-B3/USA/12R3/2016, complete genome	Human	adenov..	NA	45659	64909	65295	99%	0.0	99.90	35267	OR777202.1
Human adenovirus B strain human/USA/ak32_AdV3a/2004/3[P3H3F3],complete genome	Human	mastad..	NA	108098	64905	65308	99%	0.0	99.90	35264	JX423380.1
Human mastadenovirus B isolate Human/China/Shanghai/3754/2011/3[P3H3F3], complet	Human	mastad..	NA	108098	64905	65287	99%	0.0	99.90	35246	MK883604.1
Human mastadenovirus B Kobe-230218 DNA, complete genome	Human	mastad..	NA	108098	64901	65186	99%	0.0	99.95	35196	LC799982.1
Human adenovirus B strain human/USA/ak34_AdV3a2/2008/3[P3H3F3], complete genome	Human	mastad..	NA	108098	64896	65282	99%	0.0	99.90	35252	JX423382.1
Human adenovirus B3 strain T382/Ft Jackson South Carolina USA/2002, complete genome	Human	adenov..	NA	45659	64896	65269	99%	0.0	99.90	35250	KX384958.1
Human adenovirus B3 isolate BJ20170287, complete genome	Human	adenov..	NA	45659	64888	65267	99%	0.0	99.90	35236	MW748644.1
Human adenovirus B3 isolate HB20150126, complete genome	Human	adenov..	NA	45659	64857	65239	99%	0.0	99.88	35249	MW748665.1
Human adenovirus B3 isolate BJ20160214, complete genome	Human	adenov..	NA	45659	64857	65239	99%	0.0	99.88	35249	MW748641.1
Human mastadenovirus B isolate KUMC-62, complete genome	Human	mastad..	NA	108098	64812	65258	100%	0.0	99.84	35271	KY320276.1
Human mastadenovirus B isolate human/China/Shanghai/381/2004/3[P3H3F3], complete	Human	mastad..	NA	108098	64792	65206	99%	0.0	99.85	35244	MK813914.1
Human mastadenovirus B ADVB3_Fukushima_OH214_2023 DNA, nearly complete genome	Human	mastad..	NA	108098	64777	64777	99%	0.0	99.99	35091	LC823185.1
Human adenovirus B strain Guangzhou02, complete genome	Human	mastad..	NA	108098	64740	65127	99%	0.0	99.82	35269	DQ105654.4
Human adenovirus B strain Guangzhou01, complete genome	Human	mastad..	NA	108098	64728	65114	99%	0.0	99.81	35273	DQ099432.4
Human mastadenovirus B ADVB3_Fukushima_O573_2019 DNA, complete genome	Human	mastad..	NA	108098	64705	64705	99%	0.0	99.95	35090	LC817371.1
Human mastadenovirus B ADVB3_Fukushima_H739_2019 DNA, nearly complete genome	Human	mastad..	NA	108098	64704	64704	99%	0.0	99.95	35088	LC703523.1
Human adenovirus B3 isolate HAdV-B3/USA/7D8/2010, complete genome	Human	adenov..	NA	45659	64667	64667	99%	0.0	99.97	35049	OR876397.1
Human mastadenovirus B Kobe-230147 DNA, complete genome	Human	mastad..	NA	108098	64644	64644	99%	0.0	99.96	35049	LC799978.1
Human mastadenovirus B Nara-A230067 DNA, complete genome	Human	mastad..	NA	108098	64641	64641	99%	0.0	99.96	35050	LC799993.1
Human mastadenovirus B Nara-A230084 DNA, complete genome	Human	mastad..	NA	108098	64635	64635	99%	0.0	99.95	35050	LC799997.1
Human mastadenovirus B Kobe-230174 DNA, complete genome	Human	mastad..	NA	108098	64632	64632	99%	0.0	99.96	35046	LC799979.1
Human adenovirus B3 isolate BJ20180581, complete genome	Human	adenov..	NA	45659	64632	64632	99%	0.0	99.95	35053	MW748652.1
Human adenovirus B3 isolate BJ20180681, complete genome	Human	adenov..	NA	45659	64620	64620	99%	0.0	99.95	35052	MW748655.1
Human adenovirus B3 isolate BJ20180274, complete genome	Human	adenov..	NA	45659	64596	64596	99%	0.0	99.93	35057	MW748649.1
Human adenovirus B strain human/USA/MEEI_00075/X/3[P3H3F3],complete genome	Human	mastad..	NA	108098	64447	64839	100%	0.0	99.66	35264	KF268202.1
Human adenovirus B strain human/USA/CL_45/1988/3[P3H7F3],complete genome	Human	mastad..	NA	108098	64441	64835	100%	0.0	99.66	35265	KF268132.1
Human adenovirus B strain human/USA/CL_46/1988/3[P3H3F3], complete genome	Human	mastad..	NA	108098	64419	64818	100%	0.0	99.65	35264	KF268128.1
Human adenovirus type 3 strain NHRC 1276, complete genome	Human	adenov..	NA	45659	64364	64763	100%	0.0	99.62	35265	AY599836.1
Human mastadenovirus B Kobe-230143 DNA, complete genome	Human	mastad..	NA	108098	64360	64759	100%	0.0	99.61	35269	LC799977.1
Human adenovirus B strain human/USA/UFL_Adv3a17/2007/3[P3H3F3], complete genome	Human	mastad..	NA	108098	64356	64754	100%	0.0	99.61	35263	KF268131.1
Human mastadenovirus B isolate DW-RAT-049 genome assembly, chromosome: 1	Human	mastad..	NA	108098	64355	64741	99%	0.0	99.61	35269	OY757596.1
Human mastadenovirus B isolate DW-RAT-101 genome assembly, chromosome: 1	Human	mastad..	NA	108098	64355	64741	99%	0.0	99.61	35263	OY757647.1
Human mastadenovirus B Kobe190239 DNA, complete genome	Human	mastad..	NA	108098	64353	64752	100%	0.0	99.61	35268	LC791162.1
Human mastadenovirus B Kobe190174 DNA, complete genome	Human	mastad..	NA	108098	64353	64752	100%	0.0	99.61	35267	LC791154.1
Human mastadenovirus B isolate DW-RAT-003 genome assembly, chromosome: 1	Human	mastad..	NA	108098	64353	64739	99%	0.0	99.61	35259	OY757691.1
Human mastadenovirus B Kobe190439 DNA, complete genome	Human	mastad..	NA	108098	64347	64746	100%	0.0	99.61	35267	LC791173.1
Human mastadenovirus B Kobe190014 DNA, complete genome	Human	mastad..	NA	108098	64347	64746	100%	0.0	99.61	35267	LC791144.1
Human mastadenovirus B Kobe180429 DNA, complete genome	Human	mastad..	NA	108098	64345	64745	100%	0.0	99.61	35266	LC791130.1
Human mastadenovirus B Kobe190388 DNA, complete genome	Human	mastad..	NA	108098	64342	64741	100%	0.0	99.61	35267	LC791168.1
Human mastadenovirus B Kobe180838 DNA, complete genome	Human	mastad..	NA	108098	64338	64737	100%	0.0	99.60	35269	LC791140.1
Human mastadenovirus B Kobe190481 DNA, complete genome	Human	mastad..	NA	108098	64332	64722	100%	0.0	99.60	35269	LC791178.1
Human mastadenovirus B human/JPN/TKYAd191191/2019/3 DNA, complete genome	Human	mastad..	NA	108098	64329	64722	100%	0.0	99.60	35266	LC695001.1
Human mastadenovirus B Kobe180510 DNA, complete genome	Human	mastad..	NA	108098	64327	64693	99%	0.0	99.61	35248	LC791133.1
Human mastadenovirus B Kobe180535 DNA, complete genome	Human	mastad..	NA	108098	64321	64709	100%	0.0	99.59	35269	LC791135.1
Human mastadenovirus B Kobe190306 DNA, complete genome	Human	mastad..	NA	108098	64314	64713	100%	0.0	99.59	35268	LC791165.1
Human mastadenovirus B Kobe180381 DNA, complete genome	Human	mastad..	NA	108098	64312	64711	100%	0.0	99.59	35271	LC791128.1
Human mastadenovirus B Kobe190165 DNA, complete genome	Human	mastad..	NA	108098	64262	64661	100%	0.0	99.57	35260	LC791153.1
Human mastadenovirus B Kobe190438 DNA, complete genome	Human	mastad..	NA	108098	64135	64534	100%	0.0	99.51	35241	LC791172.1
Human mastadenovirus B Kobe180348 DNA, complete genome	Human	mastad..	NA	108098	64129	64528	100%	0.0	99.51	35236	LC791125.1
Human mastadenovirus B Kobe180571 DNA, complete genome	Human	mastad..	NA	108098	64124	64523	100%	0.0	99.51	35242	LC791137.1
Human mastadenovirus B Kobe190194 DNA, complete genome	Human	mastad..	NA	108098	64109	64508	100%	0.0	99.50	35237	LC791159.1
Human mastadenovirus B ADVB3_Fukushima_O456_2018 DNA, complete genome	Human	mastad..	NA	108098	64020	64020	99%	0.0	99.60	35093	LC817367.1
Human mastadenovirus B ADVB3_Fukushima_O454_2018 DNA, complete genome	Human	mastad..	NA	108098	64017	64017	99%	0.0	99.60	35094	LC817366.1
Human mastadenovirus B ADVB3_Fukushima_O708_2019 DNA, complete genome	Human	mastad..	NA	108098	64011	64011	99%	0.0	99.59	35095	LC817372.1
Human mastadenovirus B ADVB3_Fukushima_O492_2018 DNA, complete genome	Human	mastad..	NA	108098	64009	64009	99%	0.0	99.59	35093	LC817368.1
Human mastadenovirus B ADVB3_Fukushima_O898_2019 DNA, complete genome	Human	mastad..	NA	108098	63954	63954	99%	0.0	99.58	35079	LC757030.1
Human mastadenovirus B ADVB3_Fukushima_O512_2019 DNA, complete genome	Human	mastad..	NA	108098	63943	63943	99%	0.0	99.56	35081	LC817369.1
Human adenovirus B3 isolate HAdV-B3/USA/2E2/2009, complete genome	Human	adenov..	NA	45659	63378	63718	99%	0.0	99.15	35199	OR753109.1
Human mastadenovirus B Nara-A230045 DNA, complete genome	Human	mastad..	NA	108098	63355	63696	99%	0.0	99.14	35199	LC799987.1
Human mastadenovirus B Nara-A230039 DNA, complete genome	Human	mastad..	NA	108098	63354	63694	99%	0.0	99.14	35197	LC799983.1
Human mastadenovirus B Kobe-230205 DNA, complete genome	Human	mastad..	NA	108098	63304	63594	99%	0.0	99.14	35170	LC799981.1
182 hits were deleted due to space reasons											
Human adenovirus type 3 strain GB, complete genome	Human	adenov..	NA	45659	52460	62360	99%	0.0	98.44	35345	AY599834.1
Human adenovirus type 7 strain Gomen, complete genome	Human	adenov..	NA	10519	51269	58612	97%	0.0	97.67	35306	AY594255.1
Human adenovirus 66 strain 87-922, complete genome	Human	adenov..	NA	1337398	60358	61086	99%	0.0	97.91	35080	JN860676.1

All hits with identity >99.8% are highlighted in green colour; the HAdV -B3, B7 and B66 prototypes in yellow

The complete document with 1000 NCBI Blast Hits and corresponding alignments is available from the authors upon request

The reference sequence HADV-B114 (OR853835.1) is highlighted in red.
