# Supplementary material for: An Epidemic of Respiratory and Ocular Infections Caused by the Reemergence of a Recombinant Human Adenovirus, the Novel Type HAdV‐B114 (P7H3F3)
Source: J Med Virol. 2025 Jun 30;97(7):e70464. doi: 10.1002/jmv.70464 (PMC12208009; doi:10.1002/jmv.70464)
Supplement: Supplementary file 3 — Supplementary Document 2 BLAST Penton revised. [file JMV-97-e70464-s001.pdf]

Supplementary document 2 BLAST Analysis Penton HAdV-B114

RID: SFGX3RPM016
Job Title:OR853835 HAdV-B114 Penton
Program: BLASTN
Database: core_nt Core nucleotide BLAST database
Query #1: OR853835 HADV-B114 Query ID: lcl|Query_1903463 Length: 1635

Description	Scientific Name	Common Name	Taxid	Max Score	Total Score	Query cover	E Value	Per. Ident	Acc. Len	Accession
Human mastadenovirus B114 isolate 43593/Han/1/2023, complete genome	Human mastad..	NA	3122028	3020	3020	100%	0.0	100.00	35262	OR853835.1
Human adenovirus B strain human/USA/UFL_Adv3a51/2007/3[P3H3F3], complete genome	Human mastad..	NA	108098	3020	3020	100%	0.0	100.00	35256	KF268123.1
Human adenovirus 3 strain BJ02/CHN/2011 penton base protein gene, complete cds	Human adenov..	NA	45659	3020	3020	100%	0.0	100.00	1635	KP270907.1
Human mastadenovirus B ADVB3_Fukushima_H739_2019 DNA, nearly complete genome	Human mastad..	NA	108098	3020	3020	100%	0.0	100.00	35088	LC703523.1
Human mastadenovirus B Kobe190508 DNA, complete genome	Human mastad..	NA	108098	3020	3020	100%	0.0	100.00	35256	LC791180.1
Human mastadenovirus B Kobe-230147 DNA, complete genome	Human mastad..	NA	108098	3020	3020	100%	0.0	100.00	35049	LC799978.1
Human mastadenovirus B Kobe-230174 DNA, complete genome	Human mastad..	NA	108098	3020	3020	100%	0.0	100.00	35046	LC799979.1
Human mastadenovirus B Kobe-230181 DNA, complete genome	Human mastad..	NA	108098	3020	3020	100%	0.0	100.00	35255	LC799980.1
Human mastadenovirus B Nara-A230040 DNA, complete genome	Human mastad..	NA	108098	3020	3020	100%	0.0	100.00	35256	LC799984.1
Human mastadenovirus B Nara-A230042 DNA, complete genome	Human mastad..	NA	108098	3020	3020	100%	0.0	100.00	35254	LC799986.1
Human mastadenovirus B Nara-A230054 DNA, complete genome	Human mastad..	NA	108098	3020	3020	100%	0.0	100.00	35256	LC799989.1
Human mastadenovirus B Nara-A230056 DNA, complete genome	Human mastad..	NA	108098	3020	3020	100%	0.0	100.00	35264	LC799991.1
Human mastadenovirus B Nara-A230057 DNA, complete genome	Human mastad..	NA	108098	3020	3020	100%	0.0	100.00	35201	LC799992.1
Human mastadenovirus B Nara-A230067 DNA, complete genome	Human mastad..	NA	108098	3020	3020	100%	0.0	100.00	35050	LC799993.1
Human mastadenovirus B Nara-A230068 DNA, complete genome	Human mastad..	NA	108098	3020	3020	100%	0.0	100.00	35254	LC799994.1
Human mastadenovirus B Nara-A230077 DNA, complete genome	Human mastad..	NA	108098	3020	3020	100%	0.0	100.00	35254	LC799996.1
Human mastadenovirus B Nara-A230084 DNA, complete genome	Human mastad..	NA	108098	3020	3020	100%	0.0	100.00	35050	LC799997.1
Human mastadenovirus B ADVB3_Fukushima_O573_2019 DNA, complete genome	Human mastad..	NA	108098	3020	3020	100%	0.0	100.00	35090	LC817371.1
Human mastadenovirus B ADVB3_Fukushima_OH214_2023 DNA, nearly complete genome	Human mastad..	NA	108098	3020	3020	100%	0.0	100.00	35091	LC823185.1
Human mastadenovirus B Kobe-230228 DNA, complete genome	Human mastad..	NA	108098	3020	3020	100%	0.0	100.00	35040	LC851177.1
Human mastadenovirus B Kobe-230246 DNA, complete genome	Human mastad..	NA	108098	3020	3020	100%	0.0	100.00	35220	LC851178.1
Human mastadenovirus B Kobe-230272 DNA, complete genome	Human mastad..	NA	108098	3020	3020	100%	0.0	100.00	35220	LC851179.1
Human mastadenovirus B Kobe-230284 DNA, complete genome	Human mastad..	NA	108098	3020	3020	100%	0.0	100.00	35220	LC851180.1
Human mastadenovirus B Kobe-230327 DNA, complete genome	Human mastad..	NA	108098	3020	3020	100%	0.0	100.00	35040	LC851183.1
Human mastadenovirus B Kobe-230338 DNA, complete genome	Human mastad..	NA	108098	3020	3020	100%	0.0	100.00	35220	LC851185.1
Human mastadenovirus B Kobe-230397 DNA, complete genome	Human mastad..	NA	108098	3020	3020	100%	0.0	100.00	35050	LC851187.1
Human mastadenovirus B Nara-230094 DNA, complete genome	Human mastad..	NA	108098	3020	3020	100%	0.0	100.00	35220	LC851188.1
Human mastadenovirus B Nara-230099 DNA, complete genome	Human mastad..	NA	108098	3020	3020	100%	0.0	100.00	35040	LC851189.1
Human mastadenovirus B Nara-230115 DNA, complete genome	Human mastad..	NA	108098	3020	3020	100%	0.0	100.00	35220	LC851190.1
Human mastadenovirus B Nara-230116 DNA, complete genome	Human mastad..	NA	108098	3020	3020	100%	0.0	100.00	35255	LC851191.1
Human mastadenovirus B strain vzhadvb1, complete genome	Human mastad..	NA	108098	3020	3020	100%	0.0	100.00	35265	MH828478.1
Human mastadenovirus B isolate human/China/Shanghai/678/2009/3[P3H3F3], complete genome	Human mastad..	NA	108098	3020	3020	100%	0.0	100.00	35254	MK836308.1
Human mastadenovirus B isolate Human/China/Shanghai/3096/2009/3[P3H3F3], complete genome	Human mastad..	NA	108098	3020	3020	100%	0.0	100.00	35256	MK836311.1
Human mastadenovirus B isolate Human/China/Shanghai/3496/3[P3H3F3]/2009, complete genome	Human mastad..	NA	108098	3020	3020	100%	0.0	100.00	35255	MK847517.1
Human mastadenovirus B isolate Human/China/Shanghai/4010/2012/3[P3H3F3], complete genome	Human mastad..	NA	108098	3020	3020	100%	0.0	100.00	35254	MK883608.1
Human adenovirus B3 isolate GZ20150038 penton base protein gene	Human adenov..	NA	45659	3020	3020	100%	0.0	100.00	1635	MW748620.1
Human adenovirus B3 isolate GZ20150047 penton base protein gene	Human adenov..	NA	45659	3020	3020	100%	0.0	100.00	1635	MW748621.1
Human adenovirus B3 isolate SH20160050 penton base protein gene	Human adenov..	NA	45659	3020	3020	100%	0.0	100.00	1635	MW748622.1
Human adenovirus B3 isolate SH20160054 penton base protein gene	Human adenov..	NA	45659	3020	3020	100%	0.0	100.00	1635	MW748623.1
Human adenovirus B3 isolate WZ20150074 penton base protein gene	Human adenov..	NA	45659	3020	3020	100%	0.0	100.00	1635	MW748626.1
Human adenovirus B3 isolate WZ20150088 penton base protein gene	Human adenov..	NA	45659	3020	3020	100%	0.0	100.00	1635	MW748628.1
Human adenovirus B3 isolate WZ20150089 penton base protein gene	Human adenov..	NA	45659	3020	3020	100%	0.0	100.00	1635	MW748629.1
Human adenovirus B3 isolate HB20150330 penton base protein gene	Human adenov..	NA	45659	3020	3020	100%	0.0	100.00	1635	MW748631.1
Human adenovirus B3 isolate BJ20170260 penton base protein gene	Human adenov..	NA	45659	3020	3020	100%	0.0	100.00	1635	MW748633.1
Human adenovirus B3 isolate BJ20170262 penton base protein gene	Human adenov..	NA	45659	3020	3020	100%	0.0	100.00	1635	MW748634.1
Human adenovirus B3 isolate BJ20170268 penton base protein gene	Human adenov..	NA	45659	3020	3020	100%	0.0	100.00	1635	MW748635.1
Human adenovirus B3 isolate BJ20170306 penton base protein gene	Human adenov..	NA	45659	3020	3020	100%	0.0	100.00	1635	MW748636.1
Human adenovirus B3 isolate BJ20170365 penton base protein gene	Human adenov..	NA	45659	3020	3020	100%	0.0	100.00	1635	MW748637.1
Human adenovirus B3 isolate BJ20170371 penton base protein gene	Human adenov..	NA	45659	3020	3020	100%	0.0	100.00	1635	MW748638.1
Human adenovirus B3 isolate BJ20170380 penton base protein gene	Human adenov..	NA	45659	3020	3020	100%	0.0	100.00	1635	MW748639.1
Human adenovirus B3 isolate BJ20170320, complete genome	Human adenov..	NA	45659	3020	3020	100%	0.0	100.00	35255	MW748645.1
Human adenovirus B3 isolate BJ20170379, complete genome	Human adenov..	NA	45659	3020	3020	100%	0.0	100.00	35258	MW748646.1
Human adenovirus B3 isolate BJ20180075, complete genome	Human adenov..	NA	45659	3020	3020	100%	0.0	100.00	35255	MW748647.1
Human adenovirus B3 isolate BJ20180080, complete genome	Human adenov..	NA	45659	3020	3020	100%	0.0	100.00	35253	MW748648.1
Human adenovirus B3 isolate BJ20180567, complete genome	Human adenov..	NA	45659	3020	3020	100%	0.0	100.00	35255	MW748651.1
Human adenovirus B3 isolate BJ20180581, complete genome	Human adenov..	NA	45659	3020	3020	100%	0.0	100.00	35053	MW748652.1
Human adenovirus B3 isolate BJ20180612, complete genome	Human adenov..	NA	45659	3020	3020	100%	0.0	100.00	35280	MW748653.1
Human adenovirus B3 isolate BJ20180705, complete genome	Human adenov..	NA	45659	3020	3020	100%	0.0	100.00	35257	MW748656.1
Human adenovirus B3 isolate BJ20180708, complete genome	Human adenov..	NA	45659	3020	3020	100%	0.0	100.00	35255	MW748657.1
Human adenovirus B3 isolate BJ20180718, complete genome	Human adenov..	NA	45659	3020	3020	100%	0.0	100.00	35257	MW748658.1
Human adenovirus B3 isolate BJ20180730, complete genome	Human adenov..	NA	45659	3020	3020	100%	0.0	100.00	35271	MW748659.1
Human adenovirus B3 isolate CC20150103, complete genome	Human adenov..	NA	45659	3020	3020	100%	0.0	100.00	35257	MW748662.1
Human adenovirus B3 isolate GZ20150036, complete genome	Human adenov..	NA	45659	3020	3020	100%	0.0	100.00	35259	MW748663.1
Human adenovirus B3 isolate HB20140057, complete genome	Human adenov..	NA	45659	3020	3020	100%	0.0	100.00	35256	MW748664.1
Human adenovirus B3 isolate SH20160051, complete genome	Human adenov..	NA	45659	3020	3020	100%	0.0	100.00	35249	MW748666.1
Human adenovirus B3 isolate SH20160055, complete genome	Human adenov..	NA	45659	3020	3020	100%	0.0	100.00	35249	MW748667.1
Human adenovirus B3 isolate ZJ20150106, complete genome	Human adenov..	NA	45659	3020	3020	100%	0.0	100.00	35255	MW748670.1
Human adenovirus B3 isolate ZJ20150114, complete genome	Human adenov..	NA	45659	3020	3020	100%	0.0	100.00	35256	MW748672.1
Human adenovirus B3 isolate GZ/H201905067/2019, partial genome	Human adenov..	NA	45659	3020	3020	100%	0.0	100.00	35204	MZ540961.1
Human adenovirus B3 isolate HAdV-B3/USA/6Q2/2010, complete genome	Human adenov..	NA	45659	3020	3020	100%	0.0	100.00	35258	OQ518260.1
Human adenovirus B3 isolate HAdV-B3/USA/4R8/2009, complete genome	Human adenov..	NA	45659	3020	3020	100%	0.0	100.00	35260	OQ518276.1
Human adenovirus B3 isolate HAdV-B3/USA/7K5/2011, complete genome	Human adenov..	NA	45659	3020	3020	100%	0.0	100.00	35270	OQ518278.1
Human adenovirus B3 isolate HAdV-B3/USA/5K2/2009, complete genome	Human adenov..	NA	45659	3020	3020	100%	0.0	100.00	35262	OQ518281.1
Human adenovirus B3 isolate HAdV-B3/USA/10N2/2013, complete genome	Human adenov..	NA	45659	3020	3020	100%	0.0	100.00	35258	OQ518287.1
Human adenovirus B3 isolate HAdV-B3/USA/11D3/2013, complete genome	Human adenov..	NA	45659	3020	3020	100%	0.0	100.00	35258	OQ518292.1
Human adenovirus B3 isolate HAdV-B3/USA/7G3/2011, complete genome	Human adenov..	NA	45659	3020	3020	100%	0.0	100.00	35261	OQ518306.1
Human adenovirus B3 isolate HAdV-B3/USA/10N6/2013, complete genome	Human adenov..	NA	45659	3020	3020	100%	0.0	100.00	35254	OQ518317.1
Human adenovirus B3 isolate HAdV-B3/USA/6I2/2010, complete genome	Human adenov..	NA	45659	3020	3020	100%	0.0	100.00	35262	OQ518321.1
Human adenovirus B3 isolate HAdV-B3/USA/10Q1/2013, complete genome	Human adenov..	NA	45659	3020	3020	100%	0.0	100.00	35254	OQ518322.1
Human adenovirus B3 isolate HAdV-B3/USA/5J7/2009, complete genome	Human adenov..	NA	45659	3020	3020	100%	0.0	100.00	35268	OQ518323.1
Human adenovirus B3 isolate HAdV-B3/USA/10Q2/2013, complete genome	Human adenov..	NA	45659	3020	3020	100%	0.0	100.00	35256	OQ518344.1
Human adenovirus B3 isolate HAdV-B3/USA/1B10/2020, complete genome	Human adenov..	NA	45659	3020	3020	100%	0.0	100.00	35258	OR777156.1
Human adenovirus B3 isolate HAdV-B3/USA/6J2/2010, complete genome	Human adenov..	NA	45659	3020	3020	100%	0.0	100.00	35268	OR777168.1
Human adenovirus B3 isolate HAdV-B3/USA/6R5/2010, complete genome	Human adenov..	NA	45659	3020	3020	100%	0.0	100.00	35258	OR777169.1
Human adenovirus B3 isolate HAdV-B3/USA/12O4/2015, complete genome	Human adenov..	NA	45659	3020	3020	100%	0.0	100.00	35271	OR777196.1
Human adenovirus B3 isolate HAdV-B3/USA/12P2/2015, complete genome	Human adenov..	NA	45659	3020	3020	100%	0.0	100.00	35260	OR777198.1
Human adenovirus B3 isolate HAdV-B3/USA/16M5/2019, complete genome	Human adenov..	NA	45659	3020	3020	100%	0.0	100.00	35259	OR777235.1
Human adenovirus B3 isolate HAdV-B3/USA/7D8/2010, complete genome	Human adenov..	NA	45659	3020	3020	100%	0.0	100.00	35049	OR876397.1
Human adenovirus B3 isolate HAdV-B3/USA/9N2/2012, complete genome	Human adenov..	NA	45659	3020	3020	100%	0.0	100.00	35249	PP068614.1
Human adenovirus B3 isolate ASU101504, complete genome	Human adenov..	NA	45659	3020	3020	100%	0.0	100.00	35216	PP591781.1
Human mastadenovirus B114 isolate 24274/Han/2/2023 (P7/H3/F3),complete genome	Human mastad..	NA	3122028	3020	3020	100%	0.0	100.00	35272	PQ189736.1
Human mastadenovirus B114 isolate 26355/Han/3/2023 (P7/H3/F3),complete genome	Human mastad..	NA	3122028	3020	3020	100%	0.0	100.00	35269	PQ189737.1
Human mastadenovirus B114 isolate 38730/Han/6/2023 (P7/H3/F3),complete genome	Human mastad..	NA	3122028	3020	3020	100%	0.0	100.00	35263	PQ189739.1

Human mastadenovirus B114 isolate 51980/Han/9/2023 (P7/H3/F3),complete genome	Human	mastad..	NA	3122028	3020	3020	100%	0.0	100.00	35266	PQ189740.1
Human mastadenovirus B114 isolate 55156/Han/11/2023 (P7/H3/F3), complete genome	Human	mastad..	NA	3122028	3020	3020	100%	0.0	100.00	35283	PQ189742.1
Human mastadenovirus B114 isolate 55158/Han/12/2023 (P7/H3/F3), complete genome	Human	mastad..	NA	3122028	3020	3020	100%	0.0	100.00	35279	PQ189743.1
Human mastadenovirus B114 isolate 63735/Han/14/2023 (P7/H3/F3), complete genome	Human	mastad..	NA	3122028	3020	3020	100%	0.0	100.00	35257	PQ189745.1
Human mastadenovirus B114 isolate 66048/Han/15/2023 (P7/H3/F3), complete genome	Human	mastad..	NA	3122028	3020	3020	100%	0.0	100.00	35273	PQ189746.1
Human mastadenovirus B114 isolate 66165/Han/16/2023 (P7/H3/F3), complete genome	Human	mastad..	NA	3122028	3020	3020	100%	0.0	100.00	35267	PQ189747.1
Human mastadenovirus B114 isolate 66200/Han/18/2023 (P7/H3/F3), complete genome	Human	mastad..	NA	3122028	3020	3020	100%	0.0	100.00	35268	PQ189749.1
Human mastadenovirus B114 isolate 74412/Han/21/2023 (P7/H3/F3), complete genome	Human	mastad..	NA	3122028	3020	3020	100%	0.0	100.00	35263	PQ189751.1
Human mastadenovirus B114 isolate 48508/Han/7/2023 (P7/H3/F3), complete genome	Human	mastad..	NA	3122028	3020	3020	100%	0.0	100.00	35287	PQ189754.1
Human mastadenovirus B114 isolate 71573/Han/22/2023 (P7/H3/F3), complete genome	Human	mastad..	NA	3122028	3020	3020	100%	0.0	100.00	35279	PQ189756.1
Human mastadenovirus B L2 gene for penton base, complete cds,...	Human	mastad..	NA	108098	3014	3014	100%	0.0	99.94	1635	AB900150.1
Human adenovirus B strain human/USA/ak32_AdV3a/2004/3[P3H3F3],complete genome	Human	mastad..	NA	108098	3014	3014	100%	0.0	99.94	35264	JX423380.1
Human adenovirus B strain human/USA/ak33_AdV3a/2003/3[P3H3F3], complete genome	Human	mastad..	NA	108098	3014	3014	100%	0.0	99.94	35262	JX423381.1
Human adenovirus B strain human/USA/ak34_AdV3a2/2008/3[P3H3F3], complete genome	Human	mastad..	NA	108098	3014	3014	100%	0.0	99.94	35252	JX423382.1
Human adenovirus B strain human/USA/UFL_Adv3a2/2007/3[P3H3F3],complete genome	Human	mastad..	NA	108098	3014	3014	100%	0.0	99.94	35259	KF268120.1
Human adenovirus B strain human/USA/UFL_Adv3a50/2007/3[P3H3F3], complete genome	Human	mastad..	NA	108098	3014	3014	100%	0.0	99.94	35258	KF268133.1
Human adenovirus B strain human/USA/UFL_Adv3/2004/3[P3/H3/F3],complete genome	Human	mastad..	NA	108098	3014	3014	100%	0.0	99.94	35176	KF268195.1
Human adenovirus B strain human/CHN/Ad4/2007/NEW[P3H3F7],complete genome	Human	mastad..	NA	108098	3014	3014	100%	0.0	99.94	35265	KF268311.1
Human adenovirus 3 strain BJ19/CHN/2013 penton base protein gene	Human	adenov..	NA	45659	3014	3014	100%	0.0	99.94	1635	KP270914.1
Human adenovirus B3 strain T382/Ft Jackson South Carolina USA/2002, complete genome	Human	adenov..	NA	45659	3014	3014	100%	0.0	99.94	35250	KX384958.1
Human mastadenovirus B Kobe-230218 DNA, complete genome	Human	mastad..	NA	108098	3014	3014	100%	0.0	99.94	35196	LC799982.1
Human mastadenovirus B Kobe-230324 DNA, complete genome	Human	mastad..	NA	108098	3014	3014	100%	0.0	99.94	35270	LC851182.1
Human mastadenovirus B isolate human/China/Shanghai/1631/2009/3[P3H3F3], complete genome	Human	mastad..	NA	108098	3014	3014	100%	0.0	99.94	35255	MK836310.1
Human mastadenovirus B isolate Human/China/Shanghai/3517/2011/3[P3H3F3], complete genome	Human	mastad..	NA	108098	3014	3014	100%	0.0	99.94	35252	MK883603.1
Human mastadenovirus B isolate Human/China/Shanghai/3754/2011/3[P3H3F3], complete genome	Human	mastad..	NA	108098	3014	3014	100%	0.0	99.94	35246	MK883604.1
Human mastadenovirus B isolate P1	Human	mastad..	NA	108098	3014	3014	100%	0.0	99.94	35265	MW013769.1
Human adenovirus B3 isolate GZ20150033 penton base protein gene	Human	adenov..	NA	45659	3014	3014	100%	0.0	99.94	1635	MW748619.1
Human adenovirus B3 isolate WZ20150071 penton base protein gene	Human	adenov..	NA	45659	3014	3014	100%	0.0	99.94	1635	MW748624.1
Human adenovirus B3 isolate WZ20150072 penton base protein gene	Human	adenov..	NA	45659	3014	3014	100%	0.0	99.94	1635	MW748625.1
Human adenovirus B3 isolate WZ20150076 penton base protein gene	Human	adenov..	NA	45659	3014	3014	100%	0.0	99.94	1635	MW748627.1
Human adenovirus B3 isolate BJ20160246 penton base protein gene	Human	adenov..	NA	45659	3014	3014	100%	0.0	99.94	1635	MW748632.1
Human adenovirus B3 isolate WZ20150066, complete genome	Human	adenov..	NA	45659	3014	3014	100%	0.0	99.94	35269	MW748668.1
Human adenovirus B3 isolate WZ20150082, complete genome	Human	adenov..	NA	45659	3014	3014	100%	0.0	99.94	35251	MW748669.1
Human adenovirus B3 isolate ZJ20150111, complete genome	Human	adenov..	NA	45659	3014	3014	100%	0.0	99.94	35269	MW748671.1
Human mastadenovirus B strain Human/China/Tongliao/2019/6[P3H3F3], complete genome	Human	mastad..	NA	108098	3014	3014	100%	0.0	99.94	35256	MW767985.1
Human adenovirus B3 strain Shanxi2018-5 penton base protein gene	Human	adenov..	NA	45659	3014	3014	100%	0.0	99.94	1635	OQ128163.1
Human adenovirus B3 strain Shanxi2018-15 penton base protein gene	Human	adenov..	NA	45659	3014	3014	100%	0.0	99.94	1635	OQ128164.1
Human adenovirus B3 strain Shanxi2018-26 penton base protein gene	Human	adenov..	NA	45659	3014	3014	100%	0.0	99.94	1635	OQ128166.1
Human adenovirus B3 strain Shanxi2018-44 penton base protein gene	Human	adenov..	NA	45659	3014	3014	100%	0.0	99.94	1635	OQ128167.1
Human adenovirus B3 isolate HAdV-B3/USA/5S10/2010, complete genome	Human	adenov..	NA	45659	3014	3014	100%	0.0	99.94	35254	OQ518265.1
Human adenovirus B3 isolate HAdV-B3/USA/9A4/2012, complete genome	Human	adenov..	NA	45659	3014	3014	100%	0.0	99.94	35258	OQ518266.1
Human adenovirus B3 isolate HAdV-B3/USA/5K5/2009, complete genome	Human	adenov..	NA	45659	3014	3014	100%	0.0	99.94	35272	OQ518267.1
Human adenovirus B3 isolate HAdV-B3/USA/2E9/2009, complete genome	Human	adenov..	NA	45659	3014	3014	100%	0.0	99.94	35268	OQ518315.1
Human adenovirus B3 strain HAdV-3/China/CQ, complete genome	Human	adenov..	NA	45659	3014	3014	100%	0.0	99.94	34754	OR122655.1
Human adenovirus B3 isolate HAdV-B3_8530_May_2023, complete genome	Human	adenov..	NA	45659	3014	3014	100%	0.0	99.94	35269	OR487155.1
Human adenovirus B3 isolate HAdV-B3/USA/8T6/2012, complete genome	Human	adenov..	NA	45659	3014	3014	100%	0.0	99.94	35263	OR753127.1
Human adenovirus B3 isolate HAdV-B3/USA/9C1/2019, complete genome	Human	adenov..	NA	45659	3014	3014	100%	0.0	99.94	35270	OR777175.1
Human adenovirus B3 isolate HAdV-B3/USA/12R3/2016, complete genome	Human	adenov..	NA	45659	3014	3014	100%	0.0	99.94	35267	OR777202.1
Human mastadenovirus B114 isolate 55149/Han/10/2023 (P7/H3/F3)complete genome	Human	mastad..	NA	3122028	3014	3014	100%	0.0	99.94	35284	PQ189741.1
Human mastadenovirus B114 isolate 55162/Han/13/2023 (P7/H3/F3), complete genome	Human	mastad..	NA	3122028	3014	3014	100%	0.0	99.94	35272	PQ189744.1
Human mastadenovirus B114 isolate 66198/Han/17/2023 (P7/H3/F3), complete genome	Human	mastad..	NA	3122028	3014	3014	100%	0.0	99.94	35264	PQ189748.1
Human mastadenovirus B114 isolate 48508/Han/8/2023 (P7/H3/F3), complete genome	Human	mastad..	NA	3122028	3014	3014	100%	0.0	99.94	35276	PQ189752.1
Human mastadenovirus B114 isolate 36846/Han/5/2023 (P7/H3/F3), complete genome	Human	mastad..	NA	3122028	3014	3014	100%	0.0	99.94	35264	PQ189753.1
Human mastadenovirus B114 isolate 58834/Han/19/2023 (P7/H3/F3), complete genome	Human	mastad..	NA	3122028	3014	3014	100%	0.0	99.94	35264	PQ189755.1
Human mastadenovirus B isolate KUMC-62, complete genome	Human	mastad..	NA	108098	3009	3009	100%	0.0	99.88	35271	KY320276.1
Human mastadenovirus B isolate human/China/Shanghai/381/2004/3[P3H3F3], complete genome	Human	mastad..	NA	108098	3009	3009	100%	0.0	99.88	35244	MK813914.1
Human mastadenovirus B isolate human/China/Shanghai/538/2009/3[P3H3F3], complete genome	Human	mastad..	NA	108098	3009	3009	100%	0.0	99.88	35251	MK813915.1
Human adenovirus B3 isolate BJ20170281, complete genome	Human	adenov..	NA	45659	3009	3009	100%	0.0	99.88	35253	MW748642.1
Human adenovirus B3 isolate BJ20170284, complete genome	Human	adenov..	NA	45659	3009	3009	100%	0.0	99.88	35256	MW748643.1
Human adenovirus B3 isolate HAdV-B3/USA/7Q1/2011, complete genome	Human	adenov..	NA	45659	3009	3009	100%	0.0	99.88	35269	OQ518299.1
Human adenovirus B3 isolate HAdV-B3/USA/8E3/2011, complete genome	Human	adenov..	NA	45659	3009	3009	100%	0.0	99.88	35258	OR753121.1
Human adenovirus B strain Guangzhou02, complete genome	Human	mastad..	NA	108098	3003	3003	100%	0.0	99.82	35269	DQ105654.4
Human adenovirus B3 isolate HB20150116 penton base protein gene	Human	adenov..	NA	45659	3003	3003	100%	0.0	99.82	1635	MW748630.1
Human adenovirus B3 isolate BJ20160214, complete genome	Human	adenov..	NA	45659	3003	3003	100%	0.0	99.82	35249	MW748641.1
Human adenovirus B3 isolate BJ20180274, complete genome	Human	adenov..	NA	45659	3005	3005	100%	0.0	99.82	35057	MW748649.1
Human adenovirus B3 isolate BJ20180444, complete genome	Human	adenov..	NA	45659	3005	3005	100%	0.0	99.82	35264	MW748650.1
Human adenovirus B3 isolate BJ20180641, complete genome	Human	adenov..	NA	45659	3005	3005	100%	0.0	99.82	35264	MW748654.1
Human adenovirus B3 isolate BJ20180681, complete genome	Human	adenov..	NA	45659	3003	3003	100%	0.0	99.82	35052	MW748655.1
Human adenovirus B3 isolate BJ20180775, complete genome	Human	adenov..	NA	45659	3005	3005	100%	0.0	99.82	35262	MW748661.1
Human adenovirus B3 isolate HB20150126, complete genome	Human	adenov..	NA	45659	3003	3003	100%	0.0	99.82	35249	MW748665.1
Human adenovirus B3 strain Shanxi2018-3 penton base protein gene	Human	adenov..	NA	45659	3005	3005	100%	0.0	99.82	1638	OQ128169.1
Human adenovirus B3 strain Shanxi2018-24 penton base protein gene	Human	adenov..	NA	45659	3005	3005	100%	0.0	99.82	1638	OQ128170.1
Human adenovirus B3 strain Shanxi2018-37 penton base protein gene	Human	adenov..	NA	45659	3005	3005	100%	0.0	99.82	1638	OQ128171.1
Human adenovirus B3 strain Shanxi2018-38 penton base protein gene	Human	adenov..	NA	45659	3005	3005	100%	0.0	99.82	1638	OQ128172.1
Human adenovirus B strain Guangzhou01, complete genome	Human	mastad..	NA	108098	2998	2998	100%	0.0	99.76	35273	DQ099432.4
Human adenovirus B3 isolate BJ20170382 penton base protein gene	Human	adenov..	NA	45659	3000	3000	100%	0.0	99.76	1638	MW748640.1
Human adenovirus B3 isolate BJ20170287, complete genome	Human	adenov..	NA	45659	2998	2998	100%	0.0	99.76	35236	MW748644.1
Human adenovirus B3 isolate BJ20180734, complete genome	Human	adenov..	NA	45659	3000	3000	100%	0.0	99.76	35263	MW748660.1
Human adenovirus 66 strain 87-922, complete genome	Human	adenov..	NA	1337398	2992	2992	100%	0.0	99.69	35080	JN860676.1
Human adenovirus B strain human/USA/ak35_AdV7d2/2006/7[P7H7F7], complete genome	Human	mastad..	NA	108098	2992	2992	100%	0.0	99.69	35239	JX423383.1
Human adenovirus B strain human/ARG/ak38_AdV7h/2003/7[P7H7F7], complete genome	Human	mastad..	NA	108098	2992	2992	100%	0.0	99.69	35498	JX423386.1
Human adenovirus B strain human/USA/ak39_AdV7d2/1997/7[P7H7F7], complete genome	Human	mastad..	NA	108098	2992	2992	100%	0.0	99.69	35198	JX423387.1
Human adenovirus B strain human/USA/UFL_Adv7d2-2/unknown/7[P7H7F7], complete genome	Human	mastad..	NA	108098	2992	2992	100%	0.0	99.69	35222	KF268117.1
Human adenovirus B strain human/USA/CL_46/1988/3[P3H3F3], complete genome	Human	mastad..	NA	108098	2992	2992	100%	0.0	99.69	35264	KF268128.1
Human adenovirus B strain human/USA/CL_45/1988/3[P3H7F3],complete genome	Human	mastad..	NA	108098	2992	2992	100%	0.0	99.69	35265	KF268132.1
Human adenovirus 7 strain XY1/XY/CHN/2012, complete genome	Human	adenov..	NA	10519	2992	2992	100%	0.0	99.69	35213	KJ019880.1
Human adenovirus 7 strain K57/JM/CHN/2012, complete genome	Human	adenov..	NA	10519	2992	2992	100%	0.0	99.69	35241	KJ019882.1
Human adenovirus 7 strain K22/JM/CHN/2013, complete genome	Human	adenov..	NA	10519	2992	2992	100%	0.0	99.69	35083	KJ019883.1
Human adenovirus 7 strain H18/JM/CHN/2013, complete genome	Human	adenov..	NA	10519	2992	2992	100%	0.0	99.69	35228	KJ019886.1
Human adenovirus 7 strain L14/XY/CHN/2012, complete genome	Human	adenov..	NA	10519	2992	2992	100%	0.0	99.69	35153	KJ019888.1
Human adenovirus 7 strain HAdV-B/USA/750/1997, complete genome	Human	adenov..	NA	10519	2992	2992	100%	0.0	99.69	35242	MH910663.1
Human adenovirus 7 strain HAdV-B/USA/8010/1998, complete genome	Human	adenov..	NA	10519	2992	2992	100%	0.0	99.69	35238	MH910665.1
Human mastadenovirus B isolate SH6220	Human	mastad..	NA	108098	2992	2992	100%	0.0	99.69	35172	MN011575.1
Human adenovirus 7 strain Hunan2012-E134 penton base protein...	Human	adenov..	NA	10519	2992	2992	100%	0.0	99.69	1635	MT019929.1
Human adenovirus 7 isolate WZ059/CHN/2015, complete genome	Human	adenov..	NA	10519	2992	2992	100%	0.0	99.69	35241	MT367399.1
Human adenovirus B1 strain NICED/23-04/2283/hAdV-B/7[H7F3P7], complete genome	Human	adenov..	NA	565302	2992	2992	100%	0.0	99.69	35360	OR089145.1
Human adenovirus B1 strain NICED/23-05/2220/hAdV-B/7[H7F3P7], complete genome	Human	adenov..	NA	565302	2992	2992	100%	0.0	99.69	35331	OR089146.1
Human adenovirus B1 strain NICED/23-06/2213/hAdV-B/7[H7F3P7], complete genome	Human	adenov..	NA	565302	2992	2992	100%	0.0	99.69	35323	OR089147.1
Human adenovirus B1 strain NICED/23-08/1836/hAdV-B/7[H7F3P7],complete genome	Human	adenov..	NA	565302	2992	2992	100%	0.0	99.69	35323	OR089149.1
Human adenovirus B1 strain NICED/23-09/1639/hAdV-B/7[H7F3P7], complete genome	Human	adenov..	NA	565302	2992	2992	100%	0.0	99.69	35324	OR089150.1
Human adenovirus B1 strain NICED/23-12/2908/hAdV-B/3[H7F3P7], complete genome	Human	adenov..	NA	565302	2992	2992	100%	0.0	99.69	35341	OR130166.1

Human adenovirus B1 strain NICED/23-14/2928/hAdV-B/7[H7F3P7], complete genome	Human	adenov..	NA	565302	2992	2992	100%	0.0	99.69	35337	OR130168.1
Human adenovirus B1 strain NICED/23-15/2986/hAdV-B/7[H7F3P7], complete genome	Human	adenov..	NA	565302	2992	2992	100%	0.0	99.69	35331	OR130169.1
Human adenovirus B1 strain NICED/23-16/3234/hAdV-B/7[H7F3P7], complete genome	Human	adenov..	NA	565302	2992	2992	100%	0.0	99.69	35332	OR130170.1
Human adenovirus B1 strain NICED/23-18/3289/hAdV-B/7[H7F3P7], complete genome	Human	adenov..	NA	565302	2992	2992	100%	0.0	99.69	35332	OR130172.1
Human adenovirus B1 strain NICED/22-21/3073/hAdV-B/7[H7F3P7], complete genome	Human	adenov..	NA	565302	2992	2992	100%	0.0	99.69	35341	OR130175.1
Human adenovirus 7 isolate HAdV-B7/USA/8A10/2011, complete genome	Human	adenov..	NA	10519	2992	2992	100%	0.0	99.69	35196	OR753104.1
Human adenovirus 7 isolate HAdV-B7/USA/2D10/2009, complete genome	Human	adenov..	NA	10519	2992	2992	100%	0.0	99.69	35221	OR753139.1
Human adenovirus type 7 strain Gomen, complete genome	Human	adenov...	NA	10519	2964	2964	100%	0.0	99.39	35306	AY594255.1
Human adenovirus type 3 strain GB, complete genome	Human	adenov..	NA	45659	2887	2887	100%	0.0	98.53	35345	AY599834.1

All hits with an identity >99.8% to the whole genome of B114 (see supplementary document1) are highlighted in green colour. Despite clustering with B7 Penton many sequences are erroneously labelled as P3HxFx ".
375 hits were deleted due to space reasons. Prototypes are highlighted in yellow. The complete document with 1000 NCBI Blast Hits and corresponding alignments is available from the authors upon reques
The reference sequence HADV-B114 (OR853835.1) is highlighted in red.
